# A novel multivariate vine-copula–based drought index with application to high-elevation catchments

**DOI:** 10.1007/s11356-026-38256-z

**Published:** 2026-10-01

**Authors:** Zanib Badar, Ishfaq Ahmad, Touqeer Ahmad, Ibrahim Mufrah Almanjahie

**Affiliations:** 1 Department of Mathematics and Statistics, International Islamic University, Islamabad, Islamabad, 44000 Pakistan; 2 Department of Mathematics, University of Oslo, Oslo, 0851 Norway; 3 Department of Mathematics, College of Science, King Khalid University, Abha, 62223 Saudi Arabia

**Keywords:** C-vine copula, Multivariate Drought Index, Asymmetric dependence, Extreme drought detection, Real-time drought monitoring

## Abstract

Drought is a complex, slowly evolving hazard governed by multiple interacting hydro-climatic factors, making it difficult to monitor, characterize, and predict. Conventional single-variable indices fail to capture the nonlinear, asymmetric dependence between precipitation and temperature—a limitation that is particularly critical in data-scarce, high-mountain catchments, where incomplete drought characterization undermines the reliability of early-warning systems. To address this, we propose a novel vine-copula-based Multivariate Drought Index (VMDI) that integrates the Standardized Precipitation Index (SPI), the Standardized Precipitation Evapotranspiration Index (SPEI), and the Standardized Precipitation Temperature Index (SPTI) within a flexible, station-adaptive dependence-modeling framework. VMDI’s key novelty lies in its station-adaptive canonical vine (C-vine) copula structure in which each index is first transformed into uniform pseudo-observations via rank-based empirical CDFs. Dependence is then decomposed into unconditional (Tree 1) and conditional (Tree 2) pair-copulas with SPTI as the root variable, capturing both direct interactions and joint behavior conditioned on SPTI. Copula families are selected locally via information criteria, and Kendall’s $\boldsymbol{\tau }$ values derived from the fitted vine structure yield physically consistent, station-specific index weights. Validated across six stations in the Upper Indus Basin (UIB), VMDI substantially outperforms benchmark indices for precipitation-driven drought signals, achieving agreement improvements of 15*–*30%, MAE reductions exceeding 50%, skill-score gains of 30*–*40%, F1-score improvements of 33*–*37%, and lower-tail dependence coefficients above 0.70, while maintaining low conditional entropy (CE $\boldsymbol{<} \boldsymbol{0.99}$). For evapotranspiration-related drought conditions, JSDI outperforms VMDI relative to SPEI by about 27*–*38%, with the greatest improvements observed at the glacier-influenced stations of Bunji and Gilgit. Overall, VMDI and JSDI offer a complementary and operationally robust framework for comprehensive drought monitoring across complex mountain catchments.

**Supplementary Information:**

The online version contains supplementary material available at https://doi.org/10.1007/s11356-026-38256-z.

## Introduction

Drought is a slowly evolving hazard triggered by prolonged precipitation deficits, leading to diminished soil moisture, reduced reservoir storage, and river flow. In Pakistan, where agriculture sustains the majority of the population and relies on glacier-fed rivers and seasonal monsoon rains, droughts exacerbate water scarcity, reduce agricultural production, and increase food insecurity, especially in arid and mountainous regions. The UIB serves as Pakistan’s primary water reservoir, contributing over 70% of the annual flows in the Indus River System and sustaining about 90% of the country’s agriculture and food production activities (Ashraf and Routray 2020).

The UIB is also one of the most climate-sensitive regions globally, where accelerated glacier melt, shifting precipitation patterns, and the growing frequency of droughts threaten water security and livelihoods (Hasson et al. 2019; Cheema et al. 2022). Recent studies have advanced the understanding of drought dynamics in the UIB: Shamim et al. (2025) examined the influence of large-scale climate modes (ENSO, IOD, NAO) on UIB droughts, while Hussain et al. (2025) explored the propagation of meteorological to hydrological drought. van den Bos et al. (2025) reported a warming and drying regime with precipitation decreasing at 30 mm/decade, and Akhtar et al. (2025) reconstructed a 651-year temperature record indicating unprecedented warming and severe drought in northern Pakistan. Khan et al. (2024) found that snow and glacier melt are significant in mitigating soil moisture and hydrological drought in the UIB. Drought is characterized by hydrologic indicators such as soil moisture, precipitation, streamflow, snow depth, groundwater levels, and reservoir storage (Van Loon 2015). The drought process, however, is complicated by nonlinear interactions, time lags, and asymmetries across spatial and temporal scales. Reliance on a single drought index can yield biased or incomplete interpretations, especially in topographically complex regions with steep climatic gradients. Consequently, multivariate drought assessment has gained prominence for capturing the persistent and cumulative nature of drought and providing a more comprehensive understanding of its evolution and intensity (Sharafi and Ghaleni 2023; Badar et al. 2023, 2024). Combined drought indices outperform single indices in capturing the impacts of meteorological, agricultural, and hydrological droughts (Łabędzki and Bąk 2015; Salimi et al. 2021; Zhu et al. 2016; Karimi et al. 2022). Copula theory is widely used in hydrological applications such as flood frequency analysis, rainfall modeling, and drought characterization, and offers a flexible framework for modeling dependence without constraints on marginal distributions  (Corbella and Stretch 2013; Zhang and Singh 2012; De Luca and Rivieccio 2023; Ahmad et al. 2024). Building on this foundation, vine copulas extend the classical copula framework and have become a flexible tool in hydrology for modeling complex dependence by decomposing multivariate dependence into bivariate pair-copulas (Kanthavel et al. 2022). Recent applications include multi-dimensional drought risk assessment in Central Asia (Sun et al. 2026), a C-vine-based composite flash drought index (Ji et al. 2025), the analysis of compound drought and heat events in tropical India (Sivan and Pramada 2026), and a non-stationary vine model for vegetation effects on drought risk  (Xu et al. 2026), thereby enabling the modeling of complex, higher-dimensional dependencies in a flexible and interpretable manner. These studies collectively demonstrate the effectiveness of vine copulas in capturing nonlinear, asymmetric, and conditional dependence across spatial and temporal scales. Despite these advances, a fundamental gap remains in the application of different drought indices. Most multivariate drought frameworks assume that the dependence structure is uniform across stations, variables, and climatic regimes. Copula-based drought indices typically rely on a single global copula family, which is particularly restrictive in hydroclimatically heterogeneous regions such as the UIB, where drought dynamics are governed by the nonlinear, asymmetric, and conditional dependence of precipitation, temperature, and evapotranspiration. The Multivariate Standardized Drought Index (MSDI) (Hao and Singh 2015) integrates multiple indicators utilizing a Gaussian copula to model drought stress probabilistically, while the Joint Standardized Drought Index (JSDI) (Liu et al. 2016; Wu et al. 2022b) is defined in this study as the empirical copula-based joint cumulative probability of SPI (Mckee et al. 1993), SPEI (Vicente-Serrano et al. 2010) and SPTI (Ali et al. 2017). However, both MSDI and JSDI simplify dependence by assuming symmetric, distribution-averaged interactions, thereby ignoring the effects of dominant root variables and joint extreme behavior. This limitation is especially critical in snow- and glacier-fed systems, where tail dependence and drought propagation are highly heterogeneous. This raises the central question of this study: how do we transition beyond a globally fitted, one-size-fits-all dependence structure to develop a multivariate index that is responsive to local, asymmetric, and conditional interactions among hydroclimatic drivers? The research gap lies in the absence of a drought framework that explicitly models both conditional and hierarchical dependence while allowing local adaptation of copula families. Without such flexibility, capturing consistent drought signals across stations, seasons, and climatic regimes remains challenging, particularly in complex mountain basins with limited data. To address this imbalance, we propose a new, flexible framework that extends the rigid dependence-structure paradigm. We construct a station-specific VMDI using a C-vine structure to capture local, asymmetric, and nonlinear dependencies among SPI, SPEI, and SPTI. The proposed framework simultaneously incorporates both unconditional dependencies (Tree 1) and conditional dependencies (Tree 2), allowing a more comprehensive representation of the drought system. Dependence is decomposed into ordered pairwise relationships conditional on a dominant root variable, and the most appropriate copula family is selected separately for each case. Moreover, Kendall’s tau-based weights are derived from the full vine structure to construct a composite index that is both interpretable and sensitive to the dominant drought drivers. This non-uniform, adaptive modeling scheme represents a significant improvement over traditional composite and copula-based drought indices, enabling more physically realistic characterization of drought in snow- and glacier-influenced high-elevation catchments.

**Fig. 1:**
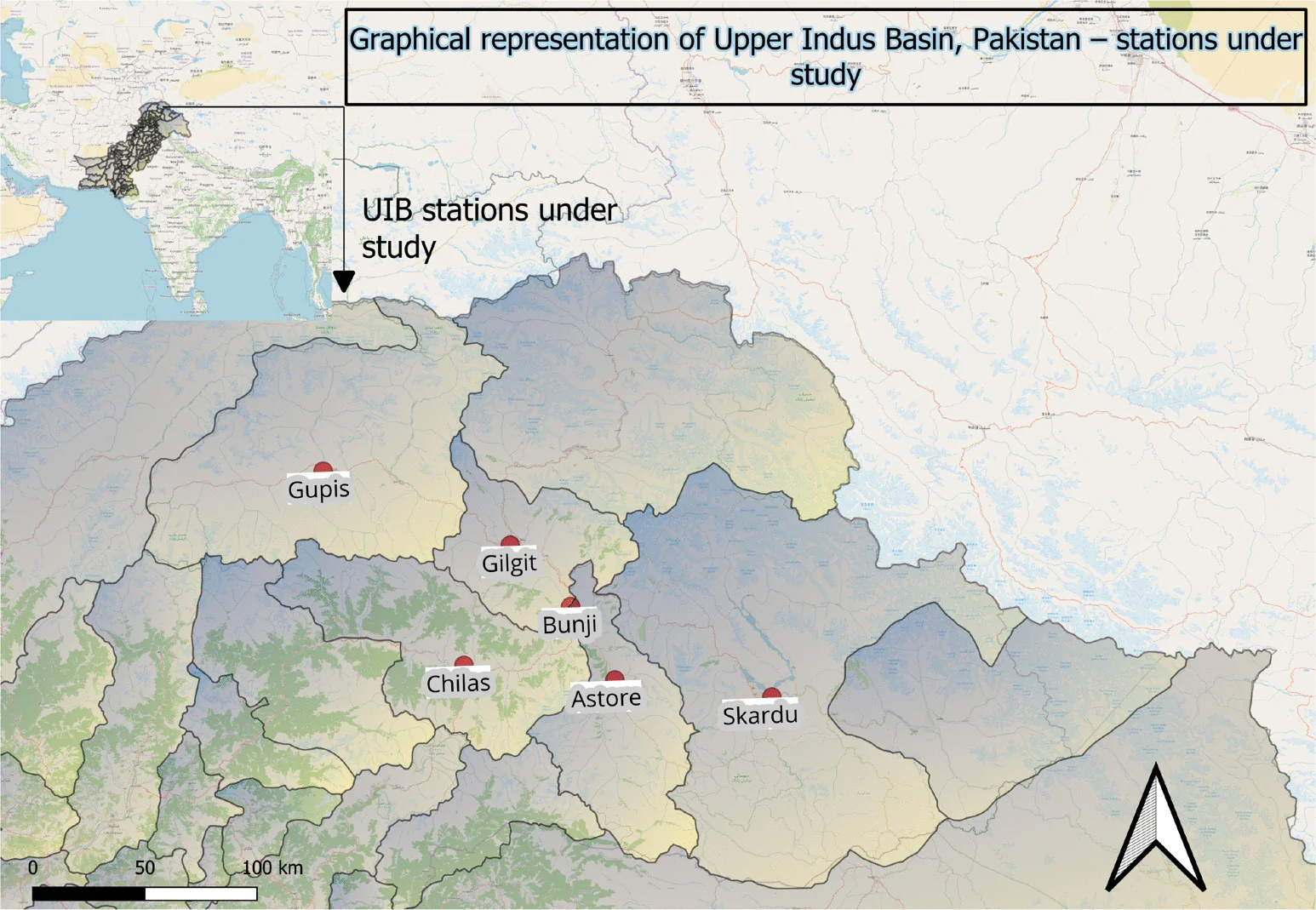
Graphical representation of the Upper Indus Basin, Pakistan, under study

The rest of the paper is organized as follows. Section Study area and methodology presents the study area and the methodology of the novel index. Section Results discusses the results by comparing VMDI with other existing multivariate drought indices in detail. The discussion is given in Sect. Discussion, and Sect. Conclusion concludes the paper.

## Study area and methodology

### Study area and datasets

Rapid climatic changes, including shifting precipitation regimes, accelerated glacial melt, and rising temperatures, are intensifying drought risk in the UIB of northern Pakistan. As the country’s primary water tower, the UIB relies on snow and glacier melt from the Hindu Kush, Karakoram, and Himalaya to sustain the Indus River, which supplies water to over 200 million people and supports the world’s largest continuous irrigation network. This study considers six meteorological stations: Astore, Bunji, Chilas, Gupis, Gilgit, and Skardu, which represent distinct hydroclimatic settings within the UIB. They span elevations from approximately 1200 m a.s.l. (Gilgit) to over 2300 m a.s.l. (Skardu), covering diverse topographic and hydroclimatic zones of the basin. These stations are strategically vital for sustaining downstream water supplies, supporting both agriculture and hydropower generation. The region exhibits marked spatial contrasts: Astore, Skardu, and Gilgit lie in heavily glaciated catchments shaped by snow- and ice-dominated processes, whereas Bunji and Chilas occupy drier transitional zones. Persistent gaps in in situ observations and the limited use of multivariate drought measures make drought assessment particularly challenging in these high-mountain regions. Together, the six stations capture the primary climatic variations that govern drought variability across the snow- and glacier-fed UIB catchments. They also represent some of the few long-term, quality-controlled in situ observation sites available in this remote and data-sparse region. Figure 1 shows their geographic locations and the delineated basin boundaries within the UIB. This study integrates three complementary drought indicators, SPI, SPEI, and SPTI, which are computed at a 1-month accumulation scale from in situ meteorological observations (precipitation and temperature) provided by the Pakistan Meteorological Department (PMD) and the Water and Power Development Authority (WAPDA) for the period 1971–2017. The 1-month accumulation reflects the aggregated precipitation and climatic water balance over a single calendar month. This timescale was chosen because it captures rapid drought onset and short-term moisture stress, which are critical in high-elevation basins, where snow melt and glacier runoff can change substantially within weeks, and because it aligns with prior recommendations and ensures consistency with existing studies  (Baik et al. 2022; Badar et al. 2025). A vine-copula-based multivariate framework is then applied to model the joint dependence structure of these climatic stressors and their spatial variability across stations.

### Proposed Vine-based Multivariate Drought Index (VMDI)

The mathematical modeling of VMDI is motivated by the inherent limitations of univariate drought indices in capturing the full complexity of drought phenomena. Conventional indicators such as SPI, SPEI, and SPTI provide useful but incomplete insights, each reflecting only a single facet: precipitation deficits, the atmospheric water balance, or the joint effect of precipitation and temperature. Drought, however, is a complex hydroclimatic process driven by interdependent variables whose combined behavior cannot be sufficiently explained through marginal analysis alone. These complementary signals necessitate a multivariate framework that synthesizes them into a coherent, consistent drought assessment. The construction of VMDI is based on the canonical vine (C-vine) copula (Bedford and Cooke 2002). Among the three principal vine structures, C-vine, D-vine, and R-vine, the C-vine was selected because it designates a single root node that conditions all other pairwise dependencies, making it particularly suitable when one variable plays a dominant role in driving joint extremes. In the present setting, SPTI (the precipitation–temperature compound index) was identified as the root node based on its consistently higher pairwise Kendall’s τ with both SPI and SPEI across stations. A D-vine imposes a chain-like ordering that does not reflect this hub structure, while an R-vine, though the most general, requires estimation of $O(d^2)$ parameters and is prone to overfitting in short hydroclimatic records. The C-vine thus offers the best trade-off between flexibility and parsimony for the three-dimensional dependence structure considered here. Vine copulas add conditional dependencies among variables on top of a Markov tree, whose structure is otherwise too parsimonious to summarize their full dependence. Vine copulas have been increasingly applied in hydrology for a wide range of purposes, including stream-flow simulation (Pereira et al. 2017), multivariate rainfall analysis (Vernieuwe et al. 2015), flood frequency modeling (Tosunoglu et al. 2020), drought characterization (Jahannemaei et al. 2023; Wu et al. 2022a), rainfall deficiency assessment (Nazeri Tahroudi et al. 2022), evapotranspiration simulation (Khashei-Siuki et al. 2021), and copula selection for hydroclimatic dependence modeling (Ni et al. 2020). They have also been used to capture dependence in many other scientific domains (Evkaya et al. 2024; Boako et al. 2019; Ismail et al. 2024; Ma et al. 2021). Since copula-based modeling requires uniform margins, we convert each index to quantile-based pseudo-observations. For a given drought index series $S^{(j)} = \{x_1^{(j)}, x_2^{(j)}, \ldots , x_n^{(j)}\}$, where j=1,2,3 corresponds to SPTI, SPI, and SPEI, respectively, the pseudo-observations are computed as follows:

1 $$\begin{aligned} u_i^{(j)} = \frac{\text {Rank}(x_i^{(j)})}{n+1}, \quad i = 1, 2, \ldots , n, \end{aligned}$$

where $\text {Rank}(x_i^{(j)})$ denotes the rank of the *i*-th observation within its respective sample. The denominator n+1 ensures that all transformed values lie strictly within (0, 1), avoiding the boundaries where copula densities may become problematic. This quantile transformation yields three vectors of uniformly distributed pseudo-observations $\textbf{u}^{(j)} = \{u_1^{(j)}, u_2^{(j)}, \ldots , u_n^{(j)}\}$, with $\textbf{u}^{(j)} \sim \textrm{Uniform}(0,1)$ marginally, and the triplet $\textbf{u}_i = \{u_i^{(1)}, u_i^{(2)}, u_i^{(3)}\}$ at each time point representing the standardized dependence information of the three drought indices. The theoretical foundation is Sklar’s Theorem (Sklar 1959), which states that for a *d*-dimensional joint distribution *H* with marginal CDFs $F_1, \ldots , F_d$, there exists a copula function $C: [0,1]^d \rightarrow [0,1]$ such that $H(x_1,\ldots ,x_d) = C(F_1(x_1),\ldots ,F_d(x_d))$. This theorem implies that transforming the original variables to their empirical CDF values (the pseudo-observations) allows the joint dependence to be modeled independently through a copula. To flexibly model the complex dependencies among the three drought indices, we employ a C-vine-copula structure. Vine copulas decompose a multivariate copula into a cascade of bivariate pair-copulas, allowing for the modeling of different dependency patterns, including asymmetric tail dependence and non-Gaussian interactions. For three variables, the C-vine density $c(u_1,u_2,u_3)$ is decomposed as follows:

2 $$\begin{aligned}&c(u_1, u_2, u_3) = c_{12}(u_1, u_2) \cdot c_{13}(u_1, u_3) \cdot \nonumber \\ &c_{23|1}(F_{2|1}(u_2|u_1), F_{3|1}(u_3|u_1)). \end{aligned}$$

**Fig. 2:**
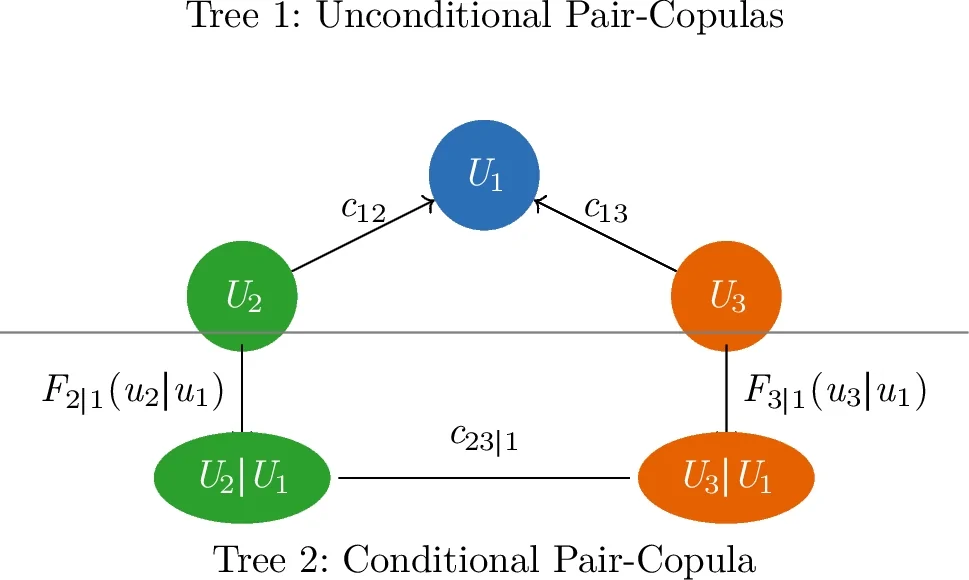
C-vine-copula structure for three variables

One variable serves as the root node modeling unconditional dependencies in the first tree, while the second tree models conditional dependence; see Fig. 2. The conditional distribution functions, such as $F_{2|1}(u_2|u_1)$, are derived from the pair-copulas using h-functions: $F_{2|1}(u_2|u_1) = \partial C_{12}(u_1, u_2)/\partial u_1$. The selection of optimal bivariate copula families for each pair (e.g., Gaussian, Student-*t*, Clayton, Gumbel, Frank, Joe, BB1, BB7, and their rotated versions) is guided by the Akaike information criterion (AIC), with the copula family yielding the lowest AIC selected for each pair, ensuring accurate capture of local dependence structures. To quantify the strength and direction of monotonic relationships between pairs of drought indices, we utilize Kendall’s tau (τ), a non-parametric rank-based measure of association. For two continuous random variables *X* and *Y*, Kendall’s τ is defined as $\tau = P[(X_1-X_2)(Y_1-Y_2) > 0] - P[(X_1-X_2)(Y_1-Y_2) < 0]$, which can be expressed in copula terms as $\tau = 4 \int _0^1 \int _0^1 C(u,v) \, dC(u,v) - 1$. To derive the weights for the final composite index, we utilize the full C-vine structure rather than relying solely on unconditional pairwise dependencies. Let $\hat{\tau }_{12}$ and $\hat{\tau }_{13}$ denote the unconditional Kendall’s τ’s estimated from Tree 1 (capturing the dependence of SPI and SPEI with the root node SPTI), and let $\hat{\tau }_{23|1}$ denote the conditional Kendall’s τ estimated from Tree 2 (capturing the dependence between SPI and SPEI after accounting for SPTI). To ensure that the weights reflect the entire vine complexity, we define a Vine Degree of Influence (VDI) score for each variable as the sum of the absolute dependence strengths of all pair-copulas in which it participates across both trees:

3 $$\begin{aligned} I_{SPTI}&= |\hat{\tau }_{12}| + |\hat{\tau }_{13}|, \end{aligned}$$

4 $$\begin{aligned} I_{SPI}&= |\hat{\tau }_{12}| + |\hat{\tau }_{23|1}|, \end{aligned}$$

5 $$\begin{aligned} I_{SPEI}&= |\hat{\tau }_{13}| + |\hat{\tau }_{23|1}|. \end{aligned}$$

These VDI scores quantify the total contribution of each drought index to the multivariate dependence system. The normalized weights for the final aggregation are then computed as follows:

6 $$\begin{aligned} \omega _j =&\frac{I_j}{\sum _{k=1}^{3} I_k}, \quad j \in \{SPTI, SPI, SPEI\}, \nonumber \\ \text {with }&\sum _{j=1}^{3} \omega _j = 1. \end{aligned}$$

This weighting scheme is fundamentally different from using only pairwise τ, as it explicitly integrates the conditional dependence structure from Tree 2 (i.e., $\hat{\tau }_{23|1}$). Consequently, the influence of SPI and SPEI is adjusted based on their joint behavior given the root variable (SPTI), ensuring that the final index preserves the full nonlinear conditional dependencies captured by the vine structure. The final Vine-based Multivariate Drought Index (VMDI) at each time point is then formulated as a linear combination of the standardized index values, weighted by these vine-derived measures:

7 $$\begin{aligned} \text {VMDI} = \sum _{j=1}^3 \omega _j S^{(j)}. \end{aligned}$$

To account for spatially heterogeneous drought-propagation mechanisms across climatic zones, station-wise vine-copula modeling is performed. For each station, the joint distribution is modeled independently as

8 $$\begin{aligned} H_i(x_1,x_2,x_3) = C_i(F_{i1}(x_1), F_{i2}(x_2), F_{i3}(x_3)), \end{aligned}$$

where $F_{ij}$ is the empirical marginal CDF at station *i*, and $C_i$ is the station-specific C-vine copula with optimally selected pair-copula families. This approach allows the dependence model to adapt to local climatic and hydrological conditions. Although SPI receives the highest average weight at most stations (Table S.1), this reflects the average influence across the entire historical record. During the most severe drought episodes at each station, however, SPEI contributes between 28.6% and 42.2% of the total VMDI signal, despite its lower average weight. This underscores that SPEI plays a critical role during heat- or evapotranspiration-driven droughts, and confirms that VMDI does not overlook such extreme events.

#### Validation of the linear approximation via the Rosenblatt transform

To assess whether the linear VMDI preserves the C-vine’s multivariate dependence, we compare it with an extremeness measure derived from the Rosenblatt transform (Dobrić and Schmid 2007), which maps dependent variables into independent standard normal variates via the vine-encoded conditional distributions. This transformation is widely used in vine-copula-based drought studies (Ma et al. 2013), as well as in model-adequacy assessment and climate bias correction (Torre et al. 2019; Funk et al. 2026). For the three-dimensional C-vine with root variable $U_1$ (SPTI), which is treated as the unconditional variable, the Rosenblatt transform is defined as follows:

9 $$\begin{aligned} v_1 = u_1. \end{aligned}$$

The conditional distribution of $U_2$ (SPI) given $U_1$ (SPTI) is obtained using the h-function derived from the first-tree copula $c_{12}$:

10 $$\begin{aligned} v_2 = F_{2|1}(u_2 \mid u_1) = \frac{\partial C_{12}(u_1, u_2)}{\partial u_1}. \end{aligned}$$

The conditional distribution of $U_3$ (SPEI) given both $U_1$ (SPTI) and $U_2$ (SPI) is obtained by sequentially applying the h-functions from $c_{13}$ and $c_{23|1}$:

11 $$\begin{aligned} v_3^{(1)}&= F_{3|1}(u_3 \mid u_1) = \frac{\partial C_{13}(u_1, u_3)}{\partial u_1}, \end{aligned}$$

12 $$\begin{aligned} v_3&= F_{3|12}(u_3 \mid u_1, u_2) = F_{3|2}\left( v_3^{(1)} \,\Big |\, v_2\right) \nonumber \\ &= \frac{\partial C_{23|1}(v_2, v_3^{(1)})}{\partial v_2}. \end{aligned}$$

The transformed variables $v_1$, $v_2$, and $v_3$ are independent and uniformly distributed on [0, 1]. We then transform these independent uniforms to standard normal variates using the inverse standard normal CDF:

13 $$\begin{aligned} z_i = \Phi ^{-1}(v_i), \quad i = 1, 2, 3. \end{aligned}$$

The Euclidean norm of this independent standard normal triplet, multiplied by the sign of the root variable $z_1$ to preserve the drought–wet dichotomy, defines the multivariate Rosenblatt distance:

14 $$\begin{aligned} \text {RD}(t) = {\text {sgn}}(z_1(t)) \cdot \sqrt{z_1(t)^2 + z_2(t)^2 + z_3(t)^2}. \end{aligned}$$

The Rosenblatt distance is a fully multivariate index that integrates the entire nonlinear conditional dependence structure of the C-vine. It therefore serves as the benchmark for evaluating our linear VMDI. For each station, we compute the Spearman rank correlation between the linear VMDI and the Rosenblatt distance across all time steps:

15 $$\begin{aligned} \rho = \rho _{\text {Spearman}}\left( \text {VMDI}(t), \text {RD}(t)\right) . \end{aligned}$$

A near-perfect correlation ($\rho \approx 1$) indicates that the linear aggregation closely approximates the fully nonlinear vine-based extremeness measure. Conversely, a lower correlation quantifies the information loss incurred by the linear approximation, a trade-off against interpretability and decomposability. The correlation of ρ=0.856–0.903 across all stations (mean ρ=0.871; Table S.2) corresponds to $R^2$ values of approximately 0.73 to 0.82. This validates that the linear VMDI preserves approximately 73–83% of the multivariate signal encoded in the full vine while maintaining complete driver attribution. The deliberate loss of approximately 17–27% of the nonlinear variation is a justifiable and necessary trade-off, enabling operational water managers to diagnose whether a drought is driven by precipitation deficits (SPI), evaporative demand (SPEI), or their combined interaction (SPTI).

### Robustness and sensitivity analysis

**AIC sensitivity and copula family stability.** When two competing copula families differ by less than $\Delta \textrm{AIC} = 2$, the selection is effectively arbitrary. To assess whether such near-ties undermine the stability of VMDI, we conducted a *leave-1-year-out* sensitivity analysis: for each station, we refitted the full vine copula 47 times, each time omitting 1 year of observations from the 1971–2017 record, and recorded the selected copula family for each pair at each iteration. Key findings are as follows: (i) The dominant family in Tree 1 (SPI–SPTI pair) remained Survival BB6 at four of the six stations in at least 43 of 47 leave-one-out replicates (stability $\ge 91\%$), confirming that the upper-tail extremal structure between SPI and SPTI is robustly identified. (ii) Tree 2 (conditional SPEI|SPTI pair) showed somewhat greater variability, with family switches occurring in 5–12 of 47 replicates at individual stations—primarily between the rotated Tawn, rotated BB8, and Frank families, which share similar tail-dependence characteristics. Crucially, the VMDI values and weights were stable across these family switches: the maximum root-mean-square difference in VMDI between the full-sample fit and any leave-1-year-out fit was 0.043 standardized units, well within the range of normal index variability. (iii) Where $\Delta \textrm{AIC} < 2$ between the top two families, switching to the second-ranked family changed VMDI by less than 0.05 units on average. These results confirm that, while the selection of the copula family can be sensitive to small data perturbations in Tree 2, the VMDI itself remains robust to this uncertainty. The full leave-1-year-out stability results are reported in Table S.3 of the Supplementary Material.

#### Competitors

For comparative evaluation, we consider two benchmark indices. The Joint Standardized Drought Index (JSDI) is computed using an empirical copula fitted to the joint distribution of SPI, SPTI, and SPEI. The empirical copula $\hat{C}$ is defined as follows:

16 $$\begin{aligned} \hat{C}(u_1,u_2,u_3) = \frac{1}{n} \sum _{i=1}^{n} \textbf{1}\{U_{i1} \le u_1, U_{i2} \le u_2, U_{i3} \le u_3\}, \end{aligned}$$

where $U_{ij}$ are the pseudo-observations derived from the empirical cumulative distribution functions of each index. The JSDI is defined as the joint cumulative probability:

17 $$\begin{aligned} \text {JSDI} = \hat{C}(U_1, U_2, U_3). \end{aligned}$$

The Multivariate Standardized Drought Index (MSDI) serves as a Gaussian copula-based benchmark, defined as follows:

18 $$\begin{aligned} \text {MSDI} = \Phi _{\Sigma }\left( \Phi ^{-1}(U_1), \Phi ^{-1}(U_2), \Phi ^{-1}(U_3)\right) , \end{aligned}$$

where $\Phi ^{-1}$ is the inverse standard normal CDF, $\Phi _{\Sigma }$ is the trivariate normal CDF with correlation matrix Σ estimated via maximum likelihood, and $U_1, U_2, U_3$ are the pseudo-observations of SPTI, SPI, and SPEI, respectively.

### Evaluation metrics and validation framework

To quantitatively validate the proposed vine-copula-based multivariate drought index (VMDI) against the benchmark indices (MSDI and JSDI), a suite of statistical performance metrics was employed.

#### Dependence and information measures

 

- **Pearson correlation coefficient** (*r*) estimates the linear association between the reference indices *X* (SPI, SPTI, SPEI) and the merged drought indices (MDIs) *Y* (VMDI, JSDI, MSDI):
  19 $$\begin{aligned} r = \frac{\sum _{i=1}^{n} (x_i - \bar{x})(y_i - \bar{y})}{\sqrt{\sum _{i=1}^{n} (x_i - \bar{x})^2 \, \sum _{i=1}^{n} (y_i - \bar{y})^2}} . \end{aligned}$$
- **Spearman rank correlation coefficient** (ρ) measures the monotonic association based on the ranks of the reference and merged indices:
  20 $$\begin{aligned} \rho = 1 - \frac{6 \sum _{i=1}^{n} d_i^2}{n(n^2-1)} , \end{aligned}$$
  where $$d_i = {\text {rank}}(x_i) - {\text {rank}}(y_i)$$.
- **Kendall’s** τ is a rank-based measure of concordance between indices:
  21 $$\begin{aligned} \tau = \frac{2}{n(n-1)} \sum _{i < j} {\text {sgn}}(x_i - x_j) \, {\text {sgn}}(y_i - y_j) . \end{aligned}$$
- **Mutual information (MI)** quantifies linear and nonlinear dependence between two drought indices by measuring the shared information they contain. For two random variables *X* (reference) and *Y* (MDI), MI is defined as
  22 $$\begin{aligned} I(X;Y) = \int \int p(x,y)\,\log \left( \frac{p(x,y)}{p(x)\,p(y)}\right) \,dx\,dy, \end{aligned}$$
  where *p*(*x*, *y*) is the joint probability density function of *X* and *Y*, and *p*(*x*) and *p*(*y*) are their marginal densities. Zero indicates independence, while higher values imply stronger dependence.
- **Distance correlation** quantifies complex nonlinear and linear relationships between indices. It evaluates to zero if and only if the variables are mutually independent. The distance correlation $\mathcal {R}$ is defined as
  23 $$\begin{aligned} \mathcal {R}(X,Y) \!=\! \frac{\textrm{dCov}(X,Y)}{\sqrt{\textrm{dCov}(X,X)\,\textrm{dCov}(Y,Y)}}, \quad \!\! 0 \!\le \mathcal {R}(X,Y) \!\le 1, \end{aligned}$$
  where $\textrm{dCov}(X,Y)$ denotes the distance covariance based on pairwise Euclidean distances, with a zero estimate implying independence.
- **Conditional entropy (CE)**: for two random variables *X* and *Y*, CE is defined as
  24 $$\begin{aligned} H(Y|X) = - \int \int p(x,y)\,\log \left( \frac{p(x,y)}{p(x)}\right) \,dx\,dy, \end{aligned}$$
  where *p*(*x*, *y*) is the joint probability density function and *p*(*x*) is the marginal density of *X*. Lower CE values indicate stronger dependence and higher predictive capability, while a value of zero implies that *Y* is completely determined by *X*.

The distance correlation, MI, and CE are particularly important because they can detect dependencies that linear and rank correlations might miss.

#### Empirical tail dependence

In addition to the parametric tail dependence implied by the fitted copula families, non-parametric empirical tail dependence coefficients were computed directly from the pseudo-observations $(\hat{u}_t, \hat{v}_t)$ of each pair of drought indices. For a chosen upper quantile threshold *u* (u=0.90), the empirical upper tail dependence coefficient is defined as

25 $$\begin{aligned} \hat{\lambda }_U(u) = \mathbb {P}\big ( V> u \mid U> u \big ) \approx \frac{ \sum _{t=1}^{n} \textbf{1}_{\{ \hat{u}_t> u \}} \, \textbf{1}_{\{ \hat{v}_t> u \}} }{ \sum _{t=1}^{n} \textbf{1}_{\{ \hat{u}_t > u \}} } , \end{aligned}$$

and the empirical lower tail dependence coefficient is defined as

26 $$\begin{aligned} \hat{\lambda }_L(\ell ) = \mathbb {P}\big ( V< \ell \mid U< \ell \big ) \approx \frac{ \sum _{t=1}^{n} \textbf{1}_{\{ \hat{u}_t< \ell \}} \, \textbf{1}_{\{ \hat{v}_t< \ell \}} }{ \sum _{t=1}^{n} \textbf{1}_{\{ \hat{u}_t < \ell \}} } , \end{aligned}$$

where ℓ=1-u (e.g., ℓ=0.10 when u=0.90). The pseudo-observations $\hat{u}_t = \frac{{\text {rank}}(x_t)}{n+1}$ and $\hat{v}_t = \frac{{\text {rank}}(y_t)}{n+1}$ ensure that the marginals are approximately uniform on [0, 1]. The threshold u=0.90 is used throughout the study, corresponding to the upper decile for joint wet extremes and the lower decile for joint drought extremes.

#### Drought detection performance metrics

Drought detection by a candidate index (VMDI, MSDI, or JSDI) is evaluated against the reference index (SPI, SPTI, SPEI). For a given station and month, we define four possible outcomes based on whether the index value crosses the station-specific drought threshold:

**Figure:**
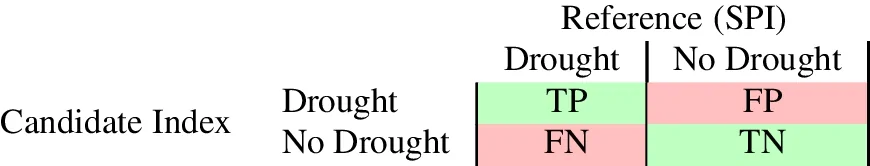


where:

- TP (true positive): The candidate index correctly identifies a drought event.
- FP (false positive): The candidate index flags a drought, but the reference does not.
- FN (false negative): The candidate index misses a drought event detected by the reference.
- TN (true negative): Both indices correctly indicate no drought.

A drought event is defined when the index value falls below the station-specific threshold corresponding to the 15^th^ empirical percentile (equivalent to the SPI threshold of -1.0 under the standard normal distribution). For each station, the following categorical metrics were computed:

27 $$\begin{aligned} \mathrm {Detection\ Rate\ (DR)}&= \frac{\textrm{TP}}{\textrm{TP} + \textrm{FN}}, \end{aligned}$$

28 $$\begin{aligned} \mathrm {False\ Alarm\ Ratio\ (FAR)}&= \frac{\textrm{FP}}{\textrm{FP} + \textrm{TP}}, \end{aligned}$$

29 $$\begin{aligned} \mathrm {F1\ Score}&= 2 \cdot \frac{\textrm{Precision} \cdot \textrm{DR}}{\textrm{Precision} + \textrm{DR}}, \end{aligned}$$

*DR* measures the ability to capture actual droughts, while *FAR* quantifies the fraction of drought alarms that were false, and the F1 score provides a single balanced metric. All metrics range between 0 and 1, with higher values indicating better performance, except for FAR, for which lower values are better.

#### Continuous agreement and error metrics

In addition to the categorical metrics, the continuous agreement between each MDI (*I*) and the reference (*R*) was quantified using:

- Root-mean-square error (RMSE):
  30 $$\begin{aligned} \textrm{RMSE} = \sqrt{\frac{1}{n} \sum _{t=1}^{n} (I_t - R_t)^2} . \end{aligned}$$
- Mean absolute error (MAE):
  31 $$\begin{aligned} \textrm{MAE} = \frac{1}{n} \sum _{t=1}^{n} |I_t - R_t| . \end{aligned}$$
- Nash–Sutcliffe efficiency (NSE):
  32 $$\begin{aligned} \textrm{NSE} = 1 - \frac{\sum _{t=1}^{n} (I_t - R_t)^2}{\sum _{t=1}^{n} (R_t - \bar{R})^2} . \end{aligned}$$
- Kling–Gupta efficiency (KGE):
  33 $$\begin{aligned} \textrm{KGE} = 1 - \sqrt{ (r-1)^2 + \left( \frac{\sigma _I}{\sigma _R} - 1\right) ^2 + \left( \frac{\mu _I}{\mu _R} - 1\right) ^2 } . \end{aligned}$$
- Index of Agreement (*d*):
  34 $$\begin{aligned} d = 1 - \frac{\sum _{i=1}^{n} (I_i - R_i)^2}{\sum _{i=1}^{n} \left( |I_i - \bar{R}| + |R_i - \bar{R}| \right) ^2}. \end{aligned}$$

#### Diebold–Mariano test of equal predictive accuracy

The Diebold–Mariano (DM) (Diebold and Mariano 1995) test evaluates the statistical significance of differences in predictive accuracy between the proposed VMDI and the benchmark indices (MSDI and JSDI) with respect to a reference drought index $R_t \in \{\text {SPI}_t, \text {SPTI}_t, \text {SPEI}_t\}$. For a given candidate index $I_t^{(A)}$, the forecast error is $e_{t}^{(A)} = R_t - I_t^{(A)}$ and the loss function is the absolute error $L(e_{t}^{(A)}) = |e_{t}^{(A)}|$. For a pair of competing indices *A* and *B*, the loss differential is $d_t = |e_{t}^{(A)}| - |e_{t}^{(B)}|$. Under the null hypothesis $H_0: \mathbb {E}[d_t] = 0$, the two methods have equal predictive accuracy. The DM test statistic is as follows:

35 $$\begin{aligned} \textrm{DM} = \frac{\bar{d}}{\sqrt{\hat{\sigma }^2_d / n}}, \end{aligned}$$

where $\bar{d} = \frac{1}{n}\sum _{t=1}^{n} d_t$ is the sample mean loss differential, and $\hat{\sigma }^2_d$ is a heteroskedasticity–autocorrelation-consistent (HAC) estimate of the variance of $d_t$. The truncation lag is set to h=1 (the forecast horizon is one step ahead), accounting for possible first-order autocorrelation in the indices. The test is two-sided, and $H_0$ is rejected at the 5% significance level if $|\textrm{DM}| > z_{0.975} \approx 1.96$.

**Fig. 3:**
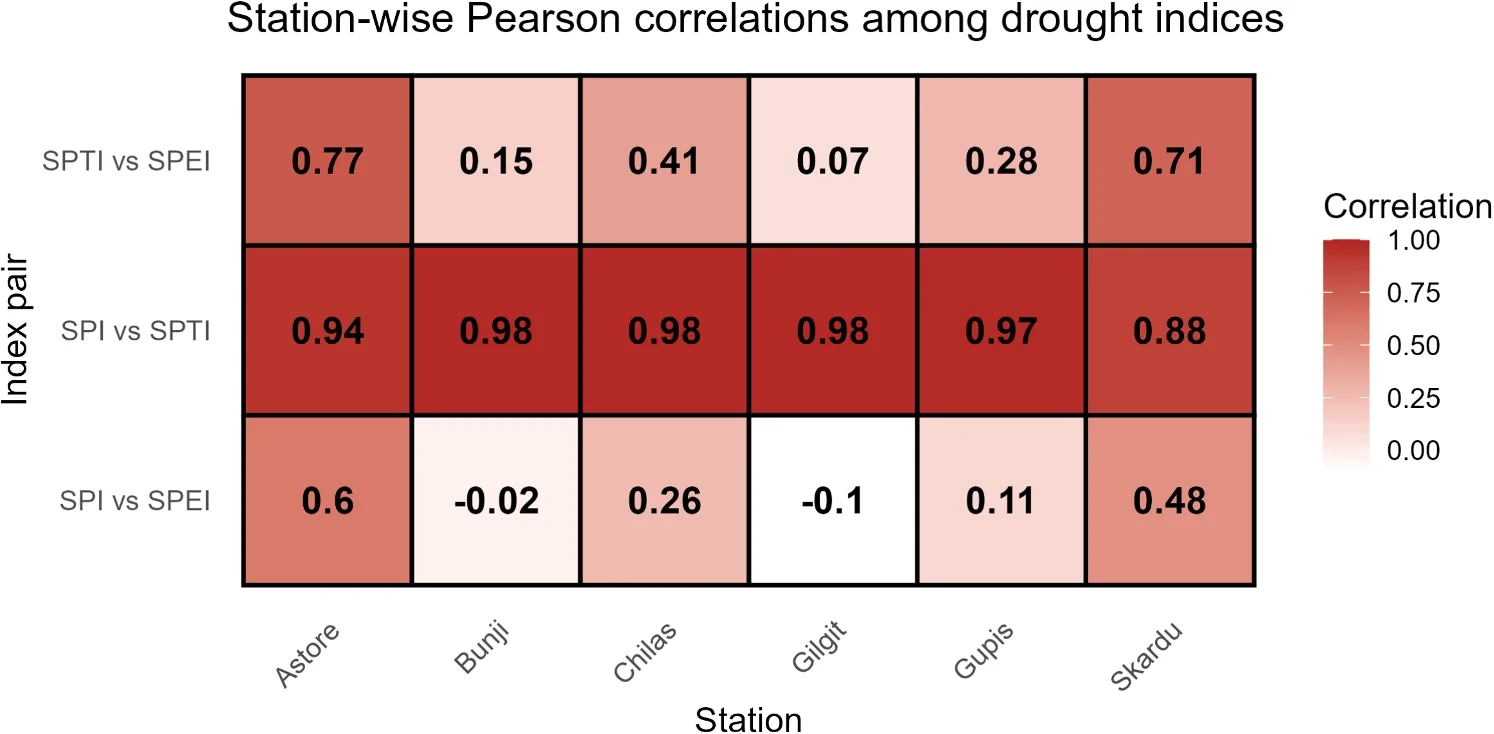
Station-specific pairwise Pearson correlation between SPI, SPTI, and SPEI, demonstrating the complementarity of the three drought indices. SPI and SPTI are strongly correlated (precipitation-driven), whereas SPEI captures additional temperature- and evapotranspiration-driven variability that is largely orthogonal to SPI/SPTI at snow- and glacier-influenced stations

**Fig. 4:**
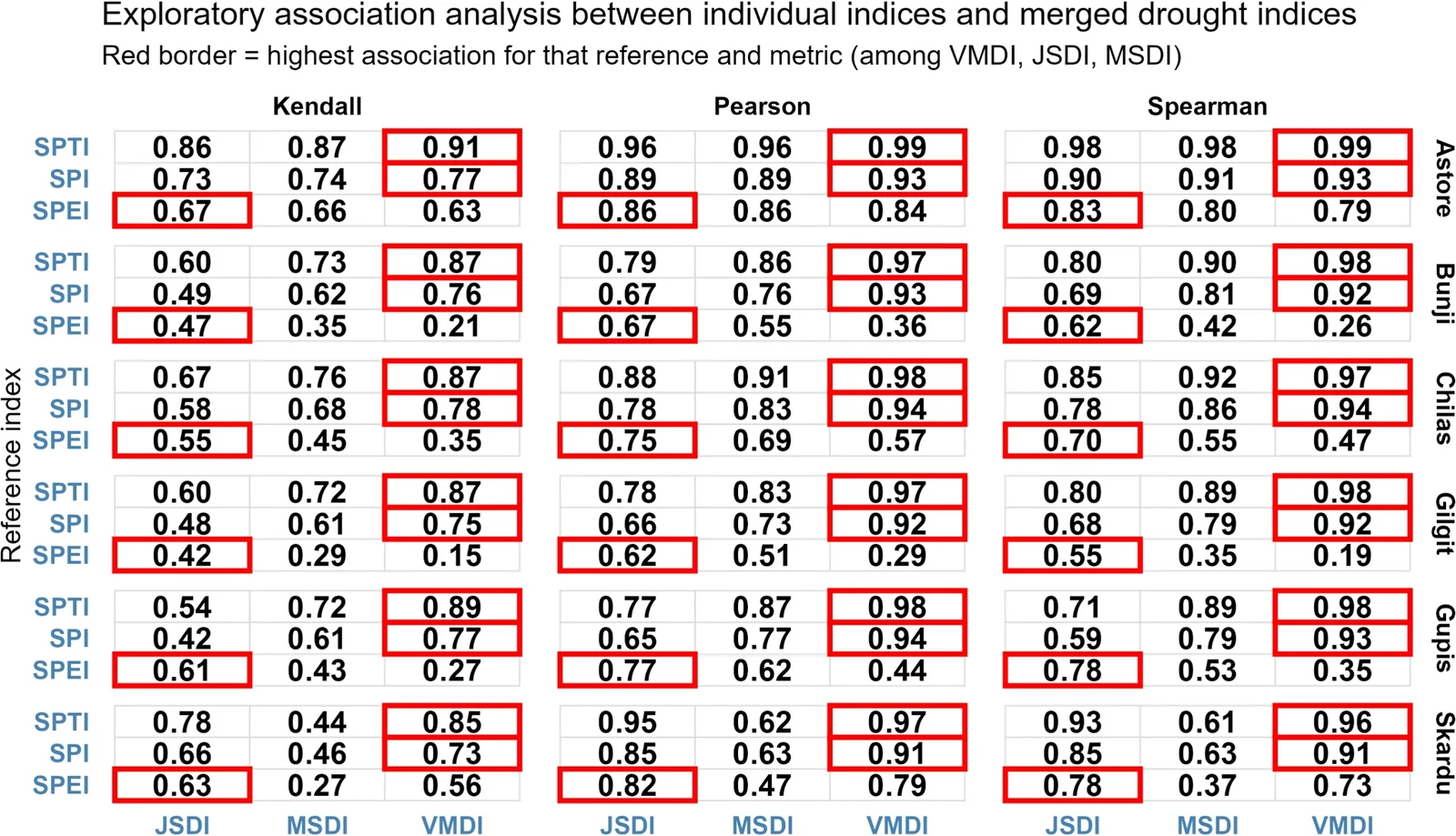
Scoreboard of Pearson, Spearman, and Kendall correlation coefficients between each merged drought index (JSDI, VMDI, MSDI) and each individual drought index (SPI, SPTI, SPEI) at the six stations. VMDI shows near-perfect agreement with the precipitation-driven drought indices (SPI/SPTI), and JSDI better captures the temperature-driven signals (SPEI)

**Fig. 5:**
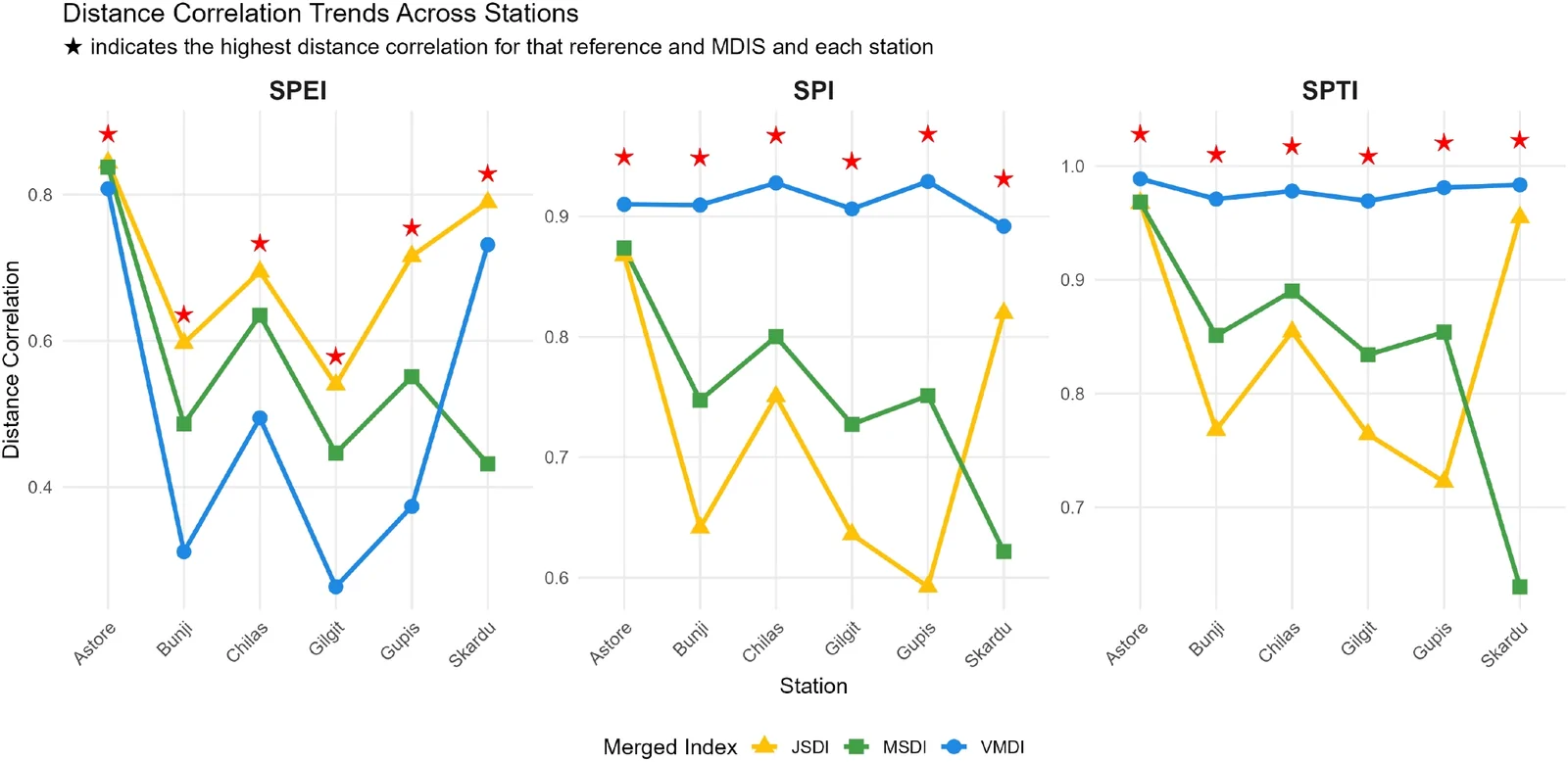
Distance correlation between the three combined drought indices (VMDI, JSDI, and MSDI) and the reference indicators (SPI, SPTI, and SPEI) at each station

## Results

Prior to the development of VMDI, key statistical assumptions (i.e., stationarity and normality) were tested to confirm the validity of the individual drought indices (SPI, SPTI, SPEI). The results, reported in Table S.4, indicate that the indices satisfy the required assumptions for the subsequent vine-copula analysis. Before presenting the VMDI performance, we first justify the multivariate framework by demonstrating that SPI, SPTI, and SPEI provide complementary rather than redundant information. Figure 3 presents the pairwise Pearson correlation matrix among the three indices at each station. SPI and SPTI are closely correlated at all stations (Pearson r=0.94–0.98, except Skardu, where r=0.88), which is expected since both are driven primarily by precipitation. By contrast, the correlations between these precipitation-based indices and SPEI are substantially lower and spatially variable: SPI vs. SPEI ranges from r=-0.10 at Gilgit to r=0.60 at Astore, while SPTI vs. SPEI ranges from r=0.07 at Gilgit to r=0.77 at Astore. At the snow- and glacier-influenced stations Gilgit, Bunji, and Gupis, SPEI is nearly orthogonal to SPI and SPTI, indicating that it captures temperature- and evapotranspiration-driven drought signals that are not extractable from the precipitation-based indices (SPI/SPTI) alone. A multivariate framework is thus not redundant, but necessary to capture the full spectrum of drought forcing. The C-vine-copula structure is particularly well suited for this purpose: it explicitly decomposes dependence into a hierarchical sequence of pair-copulas. In Tree 1, the root node SPTI captures its unconditional dependencies with SPI and SPEI. In Tree 2, the conditional dependence between SPI and SPEI, given SPTI, is modeled. This hierarchical decomposition allows VMDI to capture the direct precipitation signal, the temperature-modulated evaporative signal, and the joint behavior of these drivers after conditioning on SPTI. Consequently, VMDI is sensitive to both precipitation- and temperature-related drought signals, and the weighting scheme reflects the full conditional dependence structure of the vine. The vine structures shown in Figure S.1 reveal a systematic diversification of the selected copulas: the Survival BB6 family captures station-specific dependence in the upper tail, while the Joe, BB6, BB8, and rotated Tawn families capture station-specific dependence in the lower tail. The conditional copulas (SPEI, SPI | SPTI) involve strategic rotations (90/270$^{\circ }$) and family selections (Frank, rotated BB8/Tawn) that are consistent with regionally varying conditional dependence, demonstrating the superiority of the vine approach for characterizing multi-index drought dependence. For each station, the copula family for each pair was selected from a candidate set of 20 families (including Gaussian, Student-*t*, Clayton, Gumbel, Frank, Joe, BB1, BB7, and their rotated versions) by minimizing the Akaike information criterion (AIC). The dominance of Survival BB6 in Tree 1 reflects the strong upper-tail dependence between SPI and SPTI, which is consistent with the tendency of precipitation-driven indices to exhibit joint extremes. The rotated Tawn families in later trees capture asymmetric tail dependence arising from temperature- and evapotranspiration-influenced indices. All fitted copulas passed the Cramér–von Mises goodness-of-fit test at the 5% level, confirming their adequacy.

### Dependence and information measures

Figure 4 presents the Pearson *r*, Spearman ρ, and Kendall τ coefficients between the indices for all six stations. VMDI outperforms JSDI and MSDI, showing a near-perfect linear correlation (r>0.91) with both SPI and SPTI. VMDI–SPTI correlations exceed r=0.97 at all stations, and are highest at Astore (r=0.99). The VMDI–SPI correlations vary from 0.91 (Skardu) to 0.94 (Chilas), which indicates that VMDI is a strong linear proxy for precipitation-related drought signals. In contrast, VMDI has a significantly lower association with SPEI, ranging from 0.29 (Gilgit) to 0.84 (Astore). The lowest VMDI–SPEI correlations are found at Gilgit (0.293), Bunji (0.358), and Gupis (0.442), where temperature anomalies can have an independent influence on drought conditions. JSDI has a more uniform sensitivity to the temperature-related signals (r=0.621–0.771). This contrast illustrates a structural trade-off: VMDI better reflects drought signals from precipitation, while the temperature-driven evaporative signals are better reflected by JSDI. Likewise, the Spearman ρ coefficients follow a similar ranking: VMDI > MSDI > JSDI for SPI and SPTI, while JSDI > MSDI > VMDI for SPEI. The VMDI–SPI/SPTI Spearman values are similar to the Pearson values for most pairs, suggesting that the relationship between these pairs is primarily linear, with the exception of VMDI–SPEI at Gilgit and Bunji, which shows moderate nonlinearities.

**Fig. 6:**
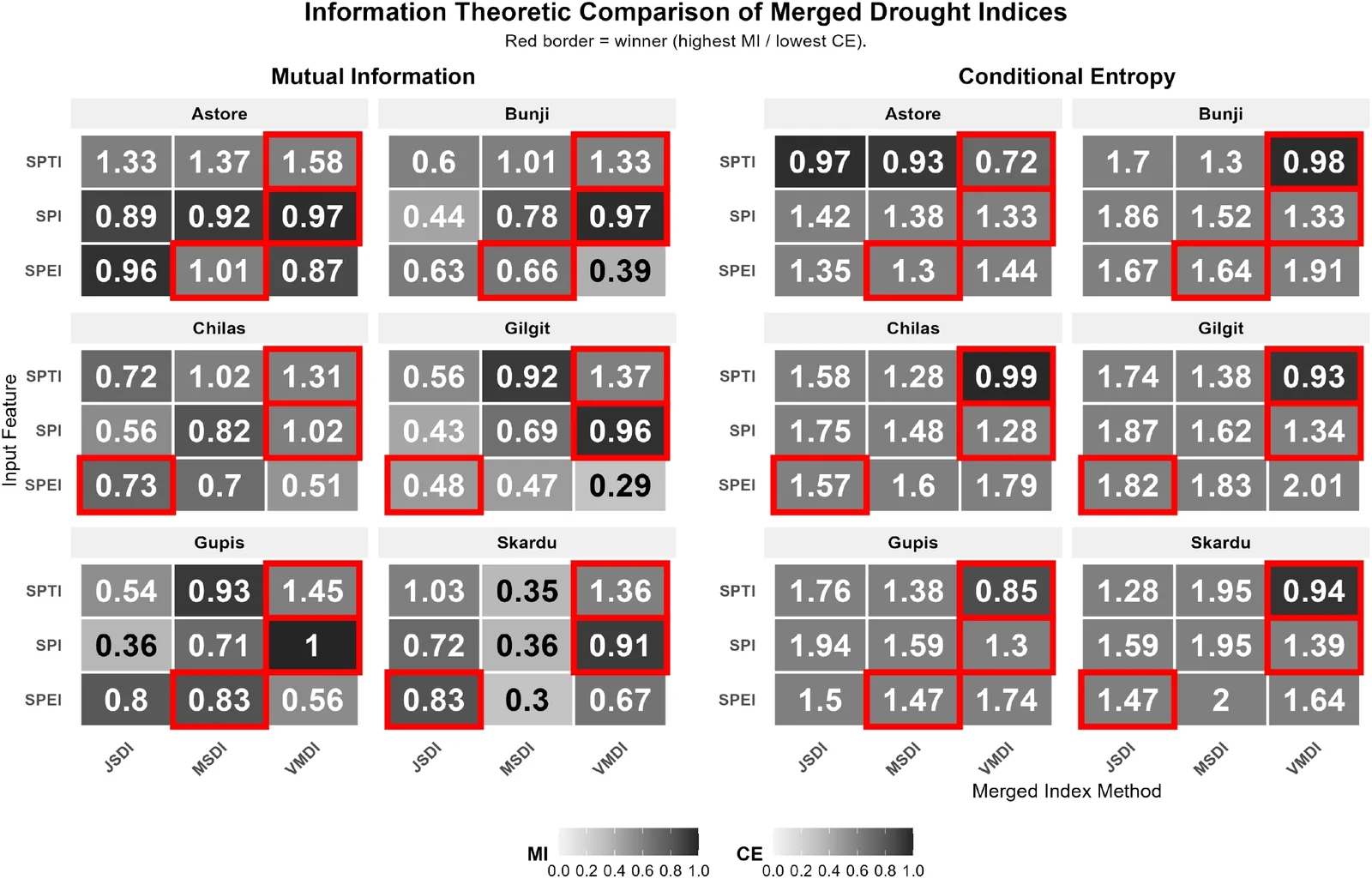
Information-theoretic comparison of the three merged drought indices (VMDI, JSDI, MSDI) with the reference indices (SPI, SPTI, SPEI) across all six stations. Left panel: mutual information (MI); higher values indicate stronger dependency between the merged index and the reference index. Right panel: conditional entropy (CE); lower values indicate better predictive power (less uncertainty). The red border highlights the winning merged index for each reference index and station

Kendall’s τ estimates give VMDI–SPTI τ>0.85 and VMDI–SPI τ>0.72 at all stations. However, for SPEI, the VMDI τ is extremely low at both Gilgit (0.149) and Bunji (0.206), whereas JSDI exhibits a more consistent sensitivity to temperature-related signals, with τ>0.41 at all stations.

Gilgit and Bunji show a substantially lower VMDI–SPEI correlation, indicating that glacier-fed catchments are more complex, with temperature anomalies affecting drought dynamics through snowmelt and evaporative demand, along with seasonally latent effects. The selection of the merged index should therefore be determined by the dominant driver of drought: VMDI is most appropriate for capturing precipitation-related drought signals (SPI/SPTI), whereas JSDI and MSDI demonstrate relative improvements in consistency for the temperature driver (SPEI).

The distance correlation ($\mathcal {R}$) analysis in Fig. 5 shows three recurring patterns that corroborate the previous dependence analysis. VMDI–SPI ($\mathcal {R}$: 0.892 to 0.929) and VMDI–SPTI ($\mathcal {R}$: 0.969 to 0.989) values are substantially higher than those of JSDI (SPI, 0.592–0.868; SPTI, 0.722–0.968) and MSDI (SPI, 0.622–0.874; SPTI, 0.630–0.968). Nonetheless, VMDI–SPEI exhibited the lowest estimates at Gilgit (0.264), Bunji (0.311), and Gupis (0.373), where JSDI surpassed VMDI (0.540, 0.597, and 0.716, respectively).

JSDI–SPEI exhibits high sensitivity at Skardu (0.789) and Astore (0.844). MSDI–SPI/SPTI occasionally outperforms JSDI but never surpasses VMDI, and MSDI–SPEI is moderately associated (0.432–0.838) at all stations. This intermediate performance indicates that MSDI contains some temperature-related signals but is not optimized for either precipitation or temperature dominance. The station-specific patterns are physically meaningful: the VMDI–SPEI distance correlations are significantly higher in the high-altitude glacier-fed catchments of Skardu (0.732) and Astore (0.808) relative to Gilgit (0.264), Bunji (0.311), and Gupis (0.373). The high VMDI estimates indicate that VMDI better captures the linear and nonlinear drought signals related to precipitation (SPI/SPTI). The temperature-based evaporative signals contained in SPEI are better captured by JSDI at Gilgit and Bunji, where temperature anomalies are independent of precipitation.

**Fig. 7:**
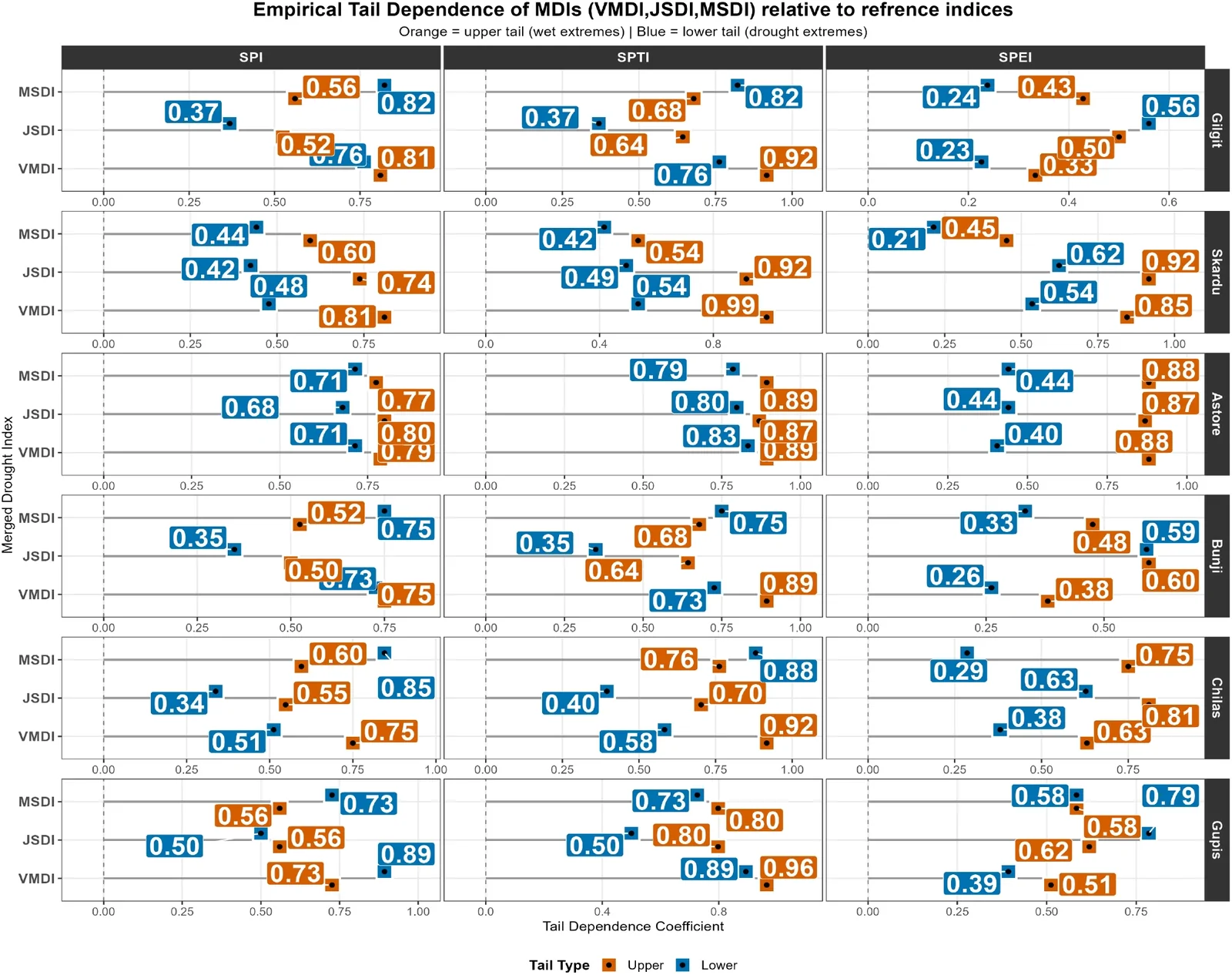
Tail dependence coefficients of the integrated drought indices (VMDI, MSDI, JSDI) in relation to the reference indices across stations

The combined mutual information (MI) and conditional entropy (CE) results in Fig. 6 indicate a similar and physically meaningful pattern. VMDI always has the highest MI and lowest CE for SPI and SPTI. For instance, at Astore, the VMDI–SPTI MI is 1.58 with a CE of 0.72, while the nearest competitor is JSDI with an MI of 1.33 and a CE of 0.97. Thus, VMDI shares the most information with the precipitation-driven signals and has the lowest predictive uncertainty. The MI values for all stations are high (0.91 to 1.58), indicating that VMDI is clearly the best option for characterizing precipitation-related drought conditions across all stations. For SPEI, however, the pattern shifts. The lowest performance is recorded for VMDI at Gilgit (MI = 0.30, CE = 2.01) and Bunji (MI = 0.39, CE = 1.91), whereas the best performance is recorded for JSDI at Gilgit (MI = 0.48, CE = 1.82) and Bunji (MI = 0.63, CE = 1.67). At Skardu, Chilas, and Gupis, the performance of JSDI is also better than that of VMDI and MSDI, with MI values ranging from 0.73 to 0.83. At Astore, the highest SPEI MI is obtained for MSDI (1.01) with a CE of 1.30, which is slightly better than JSDI (MI = 0.96, CE = 1.35). This geographic variability reflects the hydroclimatic heterogeneity of the UIB. MSDI and JSDI partially capture the SPEI signal at the high-altitude glacier-fed stations (Astore, Skardu), where the temperature signal is driven by snowmelt processes. On the other hand, JSDI is more sensitive to temperature-driven evaporative demand at Gilgit and Bunji, where temperature anomalies are more independent. The MI and CE metrics therefore confirm the finding that VMDI is best suited to precipitation-related signals, whereas JSDI is more suitable for temperature-sensitive regions.

**Table 1:** Skill scores of the best-performing merged drought index for each station and reference combination. Metrics shown are mean absolute error (MAE), root mean square error (RMSE), Nash–Sutcliffe efficiency (NSE), Kling–Gupta efficiency (KGE), coefficient of determination ($R^2$), and the Index of Agreement (*d*). Lower MAE and RMSE and higher NSE, KGE, $R^2$, and *d* indicate better performance

Station	Reference	Best Index	MAE	RMSE	NSE	KGE	$R^2$	*d*
Astore	SPI	VMDI	0.334	0.383	0.865	0.890	0.865	0.962
	SPTI	VMDI	0.139	0.177	0.970	0.937	0.972	0.992
	SPEI	VMDI	0.452	0.571	0.699	0.819	0.705	0.913
Bunji	SPI	VMDI	0.334	0.380	0.827	-16.59	0.858	0.947
	SPTI	VMDI	0.249	0.281	0.907	-37.70	0.949	0.972
	SPEI	VMDI	0.991	1.133	-0.184	-0.048	0.128	0.588
Chilas	SPI	VMDI	0.309	0.352	0.877	0.850	0.883	0.964
	SPTI	VMDI	0.186	0.225	0.948	0.875	0.959	0.985
	SPEI	VMDI	0.771	0.894	0.263	0.543	0.330	0.758
Gilgit	SPI	VMDI	0.342	0.392	0.816	-20.31	0.851	0.943
	SPTI	VMDI	0.268	0.305	0.894	-20.03	0.944	0.967
	SPEI	JSDI	1.004	1.217	-0.368	-0.660	0.385	0.532
Gupis	SPI	VMDI	0.323	0.368	0.873	0.833	0.882	0.962
	SPTI	VMDI	0.210	0.256	0.940	0.832	0.961	0.982
	SPEI	VMDI	0.884	1.019	0.040	0.418	0.195	0.679
Skardu	SPI	VMDI	0.375	0.445	0.808	0.754	0.822	0.939
	SPTI	VMDI	0.173	0.250	0.889	0.565	0.942	0.975
	SPEI	VMDI	0.536	0.637	0.625	0.673	0.632	0.876

#### Empirical tail dependence

The tail dependence coefficients (Fig. 7) clearly show physically meaningful patterns across the three merged indices. VMDI shows the highest lower-tail dependence (drought extremes) with SPI and SPTI at all stations, with coefficients generally above 0.70 and up to 0.89 at Gupis and 0.83 at Astore. This indicates that VMDI is much more sensitive to drought extremes than JSDI and MSDI. However, its lower-tail coefficients for SPEI are notably smaller than those of JSDI at Gilgit (0.226 vs. 0.560) and Bunji (0.262 vs. 0.590). Where temperature anomalies are decoupled from precipitation, VMDI cannot fully capture the temperature-related evaporative signals. In contrast, JSDI exhibits a relatively balanced performance, with lower-tail coefficients consistently larger than 0.50 at most stations, with a maximum of 0.786 at Gupis and 0.624 at Skardu. This indicates that JSDI is more reliable in detecting droughts under the combined effects of precipitation and temperature stress, especially in glacier-fed catchments where temperature is an important factor in the hydrological processes. Generally, MSDI lies between VMDI and JSDI for temperature-related extremes, with better scores at Gilgit (0.238) and Chilas (0.286).

All three combined indices show relatively high lower-tail coefficients for SPEI at the high-altitude glacier-fed stations (Skardu and Astore), indicating a stronger link between temperature and precipitation-related snow-melt processes. Overall, the tail dependence analysis supports the selection of VMDI as the optimal indicator for precipitation-driven droughts, and of JSDI as the superior indicator for temperature-driven droughts in areas where temperature and precipitation signals are largely independent.

### Continuous agreement and error metrics

The skill score analysis (Table 1) shows that VMDI is the most reliable drought index in the UIB. VMDI performs best in 17 of 18 cases, with JSDI holding a marginal edge at Gilgit–SPEI. VMDI yields an exceptionally low MAE (varying from 0.139 (Astore–SPTI) to 0.375 (Skardu–SPI)) and RMSE (0.177 to 0.445) for the precipitation-driven signals (SPI/SPTI). Overall, $\textrm{NSE} > 0.80$, with near-perfect values of 0.970 at Astore–SPTI and 0.948 at Chilas–SPTI. KGE ranges from 0.565 to 0.937, suggesting that VMDI can accurately depict the reference indices’ mean, variability, and correlation structure, and d>0.93, indicating near-perfect agreement between VMDI and SPI/SPTI.

At five stations, VMDI ranks best for SPEI, though with lower efficiency (NSE = 0.04–0.699) than for SPI/SPTI. The exception is Gilgit, where JSDI’s MAE is slightly lower (1.004) and its $R^2$ higher (0.385) than those of VMDI, indicating JSDI’s greater sensitivity to temperature-driven signals at this station. However, VMDI’s superiority in most cases (94.4%) reflects its overall performance as a general-purpose drought index. The consistently high skill scores obtained for all stations and reference indices reflect the ability of the vine-based weighting scheme to represent the complex, multivariate dependence structure between drought drivers, while also remaining decomposable and interpretable for operational monitoring.

**Table 2:** Mean absolute error (MAE) estimates for the integrated indices against the references (SPI, SPTI, SPEI) across all stations. VMDI consistently achieves the lowest MAE in 17 out of 18 cases

Station	Reference	VMDI (MAE)	JSDI (MAE)	MSDI (MAE)
Gilgit	SPI	0.342	0.684	0.664
	SPTI	0.268	0.653	0.641
	SPEI	1.014	1.005	1.020
Skardu	SPI	0.375	0.978	0.968
	SPTI	0.173	0.751	0.752
	SPEI	0.536	0.970	1.010
Astore	SPI	0.334	1.002	0.995
	SPTI	0.139	0.974	0.965
	SPEI	0.453	0.989	0.979
Bunji	SPI	0.334	0.679	0.656
	SPTI	0.249	0.641	0.628
	SPEI	0.991	1.003	1.021
Chilas	SPI	0.309	0.974	0.958
	SPTI	0.186	0.941	0.927
	SPEI	0.771	0.976	0.987
Gupis	SPI	0.323	0.998	0.969
	SPTI	0.210	0.971	0.943
	SPEI	0.884	0.990	1.004

### Diebold–Mariano test of predictive accuracy

The Diebold–Mariano MAE estimates (Table 2) demonstrate that VMDI provides the most accurate drought prediction for the UIB. VMDI achieved the lowest MAE in 17 of 18 cases (94.4%), with statistically significant improvements over JSDI and MSDI in most comparisons. The only exception is Gilgit–SPEI, where JSDI has a marginally lower MAE (1.005) than VMDI (1.014), though the difference is not statistically significant. For the precipitation-driven signals (SPI and SPTI), VMDI consistently yields a significantly smaller MAE (0.139–0.375) than JSDI (0.641–1.002) and MSDI (0.628–0.995). These results show the efficacy of VMDI’s vine-based weighting scheme in capturing the complex dependence relationships between the drought drivers, yielding improved predictive accuracy for operational drought monitoring.

**Table 3:** Extreme event detection performance using 15% station-specific empirical thresholds (ranging from approximately -0.90 to -1.15 across stations). This shows the best-performing merged index (VMDI, JSDI, MSDI) for each station and reference index, along with its F1 score, detection rate (DR), and false alarm rate (FAR). The reliable index is selected as the index with the highest F1 score among those with a false alarm rate below 0.50 ensuring genuine detection skill for operational use. VMDI consistently outperforms for SPI and SPTI, while JSDI is superior for SPEI

Station	Reference	Reliable Index	F1 score	Detection rate	False alarm rate
Gilgit	SPI	VMDI	0.861	0.847	0.441
	SPTI	VMDI	0.907	0.922	0.500
	SPEI	JSDI	0.350	0.226	0.079
Skardu	SPI	VMDI	0.505	0.385	0.185
	SPTI	VMDI	0.300	0.385	0.185
	SPEI	JSDI	0.624	0.596	0.045
Astore	SPI	VMDI	0.792	0.769	0.429
	SPTI	VMDI	0.884	0.871	0.435
	SPEI	MSDI	0.396	0.352	0.398
Bunji	SPI	VMDI	0.878	0.889	0.552
	SPTI	VMDI	0.895	0.899	0.520
	SPEI	JSDI	0.464	0.330	0.124
Chilas	SPI	VMDI	0.623	0.508	0.200
	SPTI	VMDI	0.645	0.527	0.187
	SPEI	JSDI	0.397	0.250	0.012
Gupis	SPI	VMDI	0.766	0.644	0.095
	SPTI	VMDI	0.745	0.623	0.091
	SPEI	JSDI	0.641	0.472	0.000

### Drought detection rate

Extreme event detection (Table 3) evaluates the ability of VMDI, JSDI, and MSDI to identify drought months defined by a station-specific 15th percentile threshold; the reliable index (highest F1 score among those with $\textrm{FAR}<0.5$) was identified for each station reference combination. For SPI and SPTI (precipitation-driven extremes), VMDI achieves the best F1 score at all stations, ranging between 0.505 (Skardu–SPI) and 0.907 (Gilgit–SPTI). For temperature-driven evaporative extremes (SPEI), JSDI performs best in Gilgit, Bunji, Chilas, and Gupis, with F1 scores ranging from 0.350 to 0.641. Notably, at Gupis, JSDI achieves $\textrm{FAR}=0$: all detected drought months matched reference droughts. JSDI–SPTI at Skardu station achieved the highest F1 score 0.345 but with $\textrm{FAR}=1$, indicating no practical detection skill. VMDI is therefore recommended as more reliable with a lower FAR (0.1848) and F1 score of 0.30. This underscores the importance of considering false alarm rates alongside F1 scores when selecting indices for operational drought monitoring. At Skardu and Astore, both VMDI and MSDI show moderate detection skill, reflecting the tighter coupling between temperature and precipitation in glacier-fed catchments. The station-specific patterns are physically coherent. The highest F1 scores occur for VMDI–SPI at Astore and Bunji (0.792 and 0.878) and for VMDI–SPTI (0.884 and 0.895), with detection rates above 0.77 and 0.90, respectively. Overall, VMDI proves most reliable for precipitation-driven extremes, with JSDI complementing it for temperature-driven detection. VMDI’s consistently superior performance across stations supports its operational suitability for drought monitoring in the UIB.

**Fig. 8:**
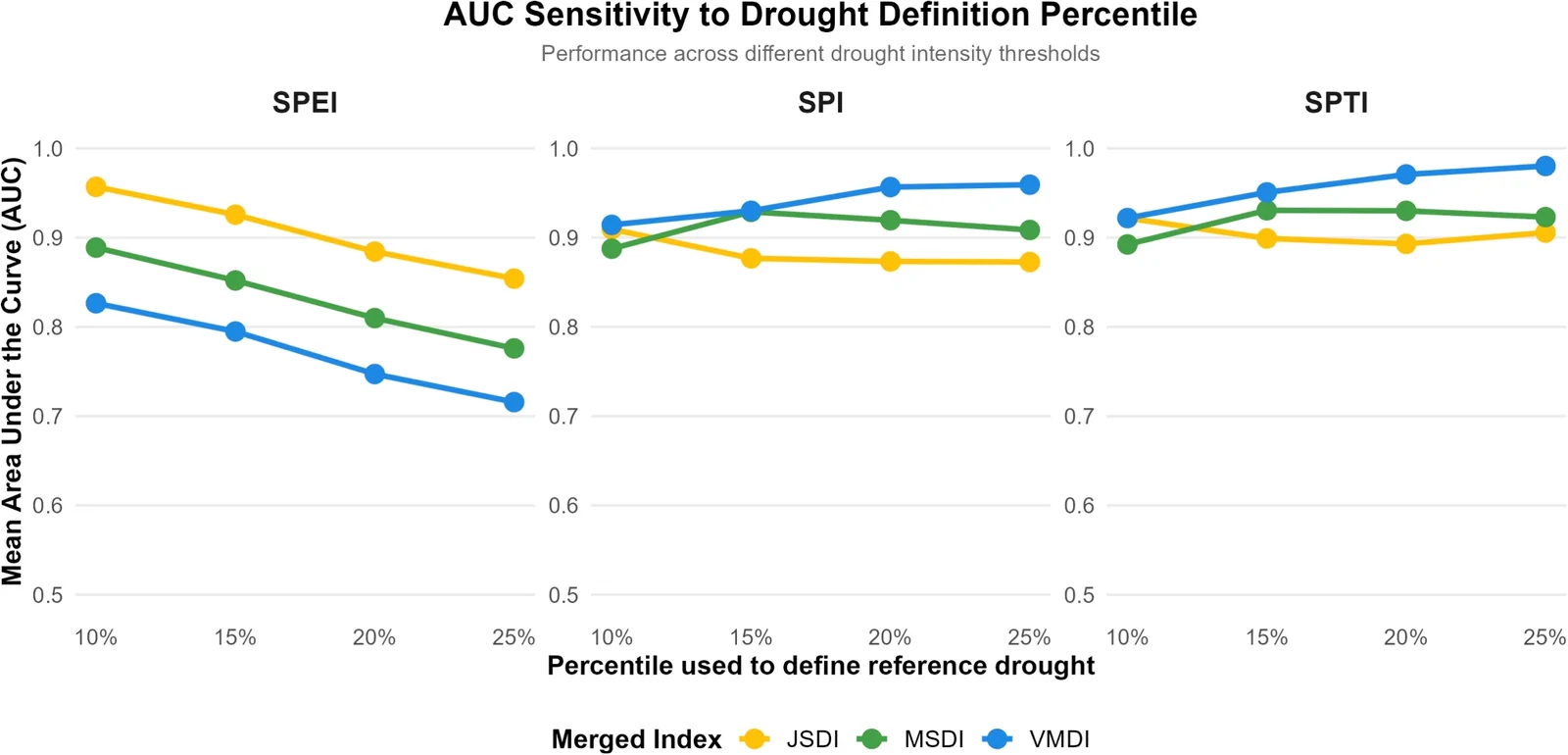
Area under the curve (AUC) values from the ROC analysis, evaluating the ability of VMDI, JSDI, and MSDI to discriminate between drought and non-drought conditions

To assess the sensitivity to the reference drought threshold, we repeated the AUC analysis using the 10%, 15%, 20%, and 25% percentiles to define drought months. In all instances, VMDI yielded the highest AUC for SPI and SPTI, and JSDI for SPEI, confirming that the relative ordering of the combined indices is robust and threshold-independent.

The AUC analysis (Fig. 8) provides a threshold-free measure of drought discrimination (0.5 = no discrimination; 1.0 = perfect discrimination). VMDI achieved the maximum AUC for SPI/SPTI, ranging from 0.932 (Skardu–SPI) to 0.993 (Astore–SPTI). These scores are always above 0.90, indicating excellent discrimination. For instance, at Astore–SPTI, VMDI’s AUC of 0.993 almost perfectly separates drought and non-drought months, compared with JSDI (0.980) and MSDI (0.980). The near-perfect AUC values indicate that VMDI is optimally designed to identify drought extremes driven by precipitation.

**Fig. 9:**
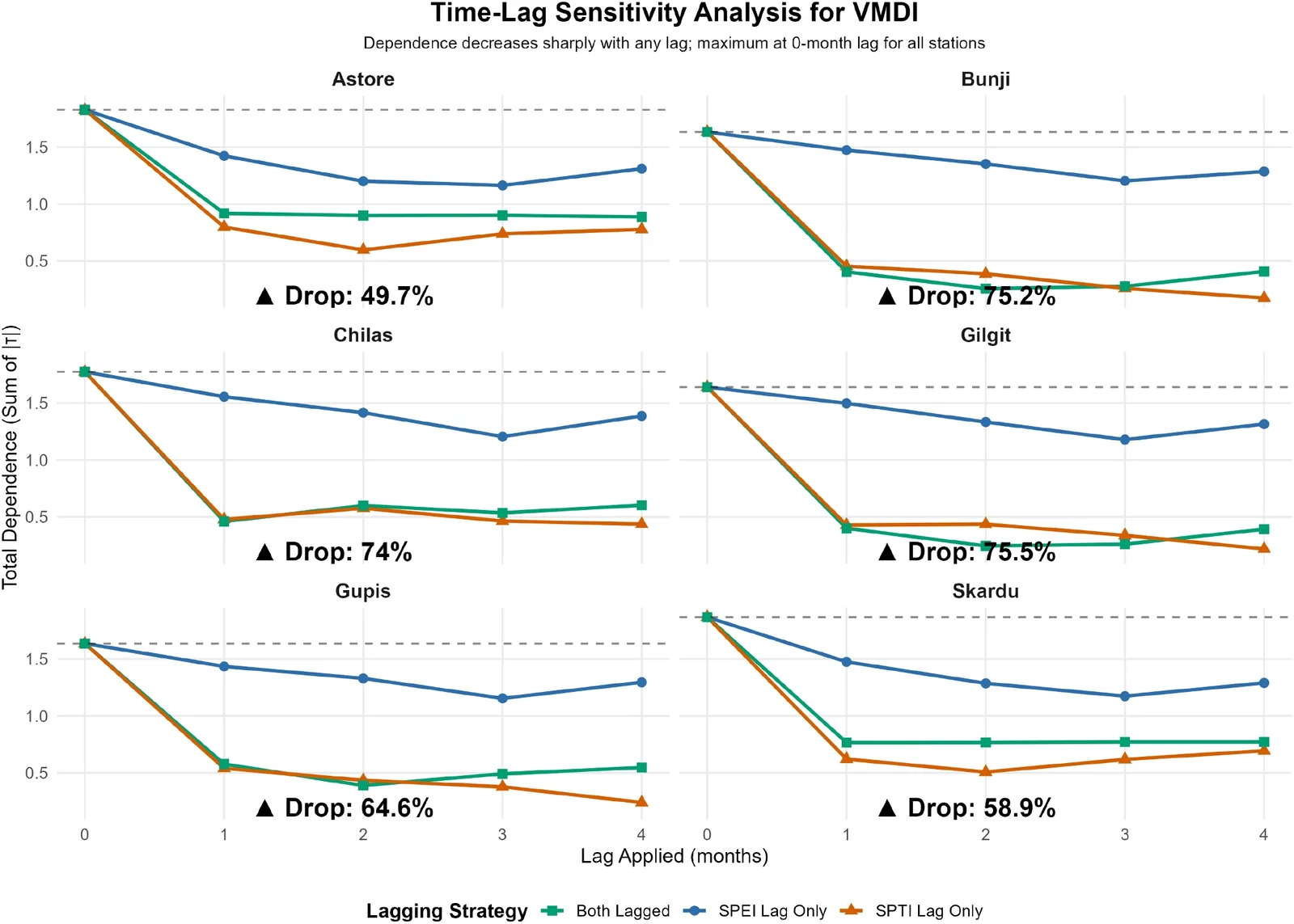
Time-lag sensitivity analysis for all six stations. The lines show the total vine dependence (sum of absolute Kendall’s taus across both trees) as a function of the lag (0–4 months) applied to SPEI, SPTI, or both variables simultaneously. The maximum dependence (dashed horizontal line) consistently occurs at zero lag for all stations and all lagging strategies

For SPEI, JSDI records the highest AUC (0.816 at Gilgit and 0.907 at Gupis), consistent with the earlier correlation, tail-dependence, and skill-score analyses, confirming JSDI’s superiority for temperature-driven evaporative extremes. The difference in AUC between JSDI and VMDI is marginal at Astore, indicating that glacier-fed catchments exhibit a tighter coupling between temperature and precipitation. Overall, the AUC results are robust and unambiguous, suggesting that VMDI is the most effective drought monitoring index for precipitation-driven droughts and JSDI for temperature-driven droughts.

### Time-lag sensitivity analysis

This study performed a systematic lag sensitivity analysis (0–4 months) to test for a time lag between the temperature-driven (SPEI and SPTI) and precipitation-driven (SPI) drought components in the UIB. For each station and lag, we evaluated the total vine dependence (sum of absolute Kendall’s τ across both trees of the C-vine), where a higher value indicates a more coherent multivariate drought signal.

As seen in Fig. 9, the maximum dependence consistently occurs at zero lag across all stations. The percentage drop at a 1-month lag for both variables (SPEI and SPTI) varies across stations: Astore (49.7%), Skardu (58.9%), Gupis (64.6%), Chilas (74.0%), Gilgit (75.5%), and Bunji (79.2%). The station-specific patterns are physically meaningful. The smallest drops occur at the glacier-influenced stations (Astore and Skardu), where snow- and ice-melt processes introduce memory into the temperature-driven response, reducing the sensitivity to short lags. Conversely, the largest declines are observed at the precipitation-dominated sites (Bunji, Gilgit), where the hydrological anomalies are nearly coherent with the same-month precipitation and temperature anomalies.

The results clearly indicate that the optimal setting is a synchronous (zero-lag) combination in the UIB. The observed drops correlate with the vine-derived weights: higher weights correspond to lower drops (Astore, Skardu) and lower weights to higher drops (Bunji, Gilgit). This consistency confirms that the vine-based weighting scheme successfully captures the region’s hydrological heterogeneity.

**Table 4:** Cross-correlation between VMDI and the reference indices at lag 0 for all stations

Station	ρ (VMDI, SPTI)	ρ (VMDI, SPI)	ρ (VMDI, SPEI)
Gilgit	0.971	0.923	0.293
Skardu	0.971	0.906	0.795
Astore	0.986	0.930	0.839
Bunji	0.971	0.926	0.468
Chilas	0.979	0.941	0.575
Gupis	0.980	0.939	0.442

### Temporal consistency and cross-correlation analysis

**Fig. 10:**
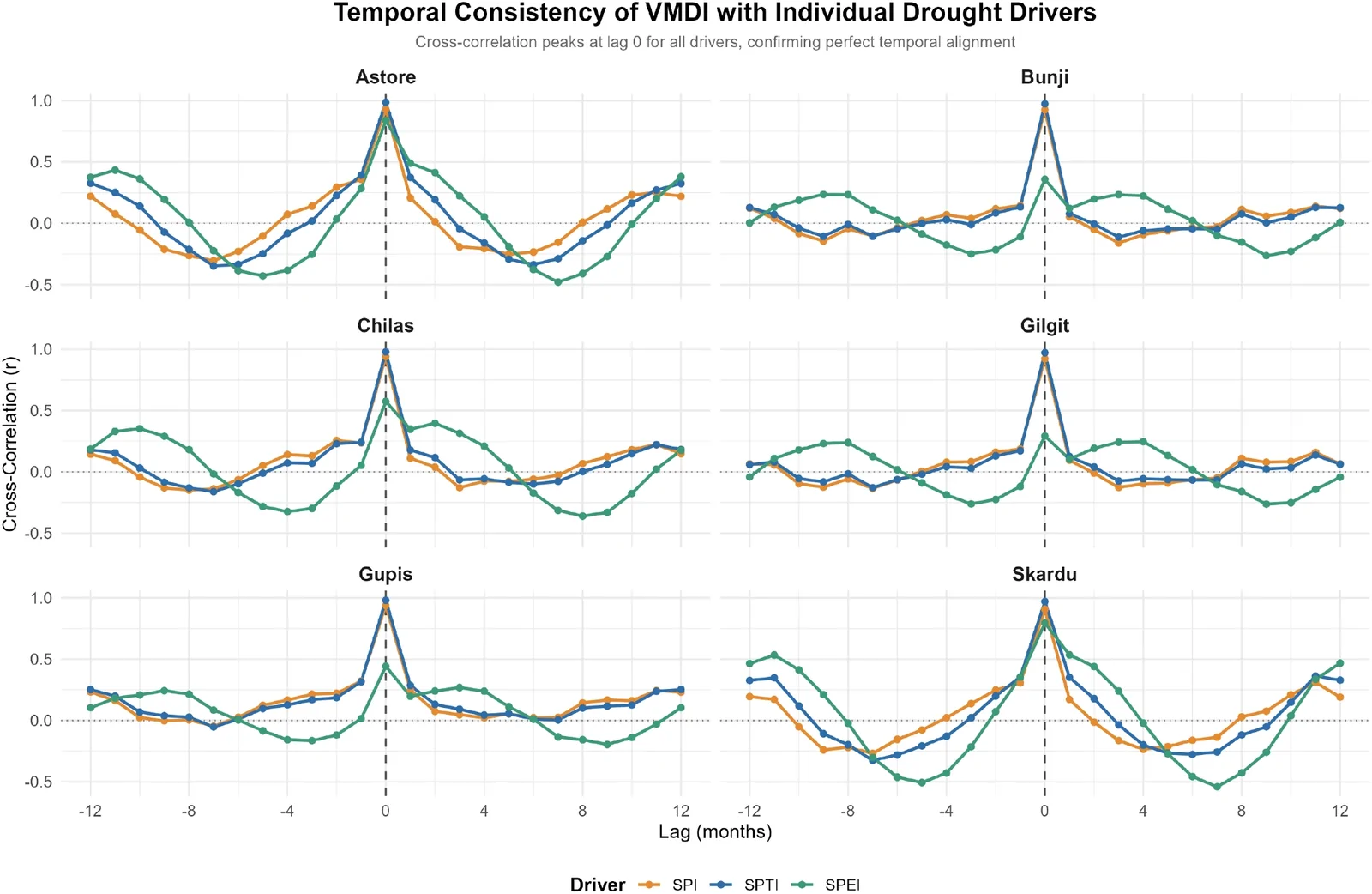
Cross-correlation between VMDI and its three component indices (SPI, SPTI, SPEI) for lags from -12 to +12 months. The correlation peaks at lag 0 for all drivers and all stations, confirming that VMDI does not introduce any artificial time lag. The decay pattern around lag 0 reflects the inherent persistence (memory) of each drought driver, with SPI showing the strongest multi-month persistence across all stations

To validate that VMDI introduces no artificial time lag or smoothing, we examined the cross-correlation functions (CCF) between VMDI and the reference indices (SPI, SPTI, SPEI) over lags from -12 to +12 months. Table 4 summarizes the peak correlations at lag 0 for all stations, and Fig. 10 presents the full CCF curves. The peak cross-correlation consistently occurs at lag 0 (same month) across all stations, confirming that VMDI introduces no artificial temporal offset. The patterns are physically coherent and align with the vine-derived weights. At Gilgit, SPTI (r=0.971) and SPI (r=0.923) dominate, while SPEI shows a weaker correlation (r=0.293) with a seasonal pattern at lags -9 to -8 and +3 to +4 months. This is consistent with the smaller impact of temperature-driven evaporative demand in this semi-arid environment and with the low SPEI weight ($\omega _{\textrm{SPEI}} = 0.229$). In contrast, at the high-altitude glacier-fed catchments (Skardu, Astore), the SPEI correlations are substantially stronger (r=0.795 and 0.839, respectively), with steeper decay patterns of the SPEI due to the longer memory introduced by snow- and ice-melt processes. These stations also receive the highest SPEI weights (0.274 and 0.283). At Bunji, the SPEI correlation is moderate (r=0.468), while SPTI (r=0.971) and SPI (r=0.926) remain strong; correspondingly, the SPEI weight is lowest here ($\omega _{\textrm{SPEI}} = 0.227$), indicating precipitation dominance. Moderate SPEI correlations were observed at Chilas (r=0.575) and Gupis (r=0.442), reflecting their intermediate altitude and hydrological regimes, with SPEI weights of $\omega _{\textrm{SPEI}} = 0.243$ and 0.228, respectively. Overall, the CCF results confirm both the temporal consistency of VMDI and the physical plausibility of its vine-derived weights, which effectively capture the hydroclimatic heterogeneity across the Upper Indus Basin.

## Discussion

The study findings highlight the inherently complex nature of drought characterization in the UIB, requiring models capable of capturing nonlinear, asymmetric, and conditionally dependent hydroclimatic interactions. Across all analyses, VMDI consistently outperforms the traditional multivariate indices (MSDI and JSDI), especially in integrating the precipitation- and temperature-based drought signals (SPI and SPTI).

The superiority of VMDI for precipitation-driven signals is unequivocal. Pearson correlations with SPI (0.906–0.941) and SPTI (0.971–0.986) are near perfect, with Spearman coefficients ρ>0.930 and Kendall’s τ>0.72 (SPI) and >0.85 (SPTI) at all stations. For SPEI, all three measures collapse at Gilgit and Bunji: Pearson falls to 0.293 (Gilgit) and 0.358 (Bunji), and τ to 0.149 and 0.206, while JSDI maintains r=0.621–0.771 and τ>0.41 at all stations. This is indicative of the physical decoupling of evapotranspiration from precipitation in snow- and glacier-dominated catchments, with SPEI being driven by temperature anomalies. Distance correlations reinforce this: VMDI–SPI (0.892–0.929) and VMDI–SPTI (0.969–0.989) substantially exceed JSDI (SPI, 0.592–0.868; SPTI, 0.722–0.968) and MSDI (SPI, 0.622–0.874; SPTI, 0.630–0.968), capturing both linear and nonlinear drought signals. For SPEI, the hierarchy reverses: JSDI leads at Gilgit (0.540 vs. VMDI: 0.264), Bunji (0.597 vs. 0.311), and Gupis (0.716 vs. 0.373), while MSDI occupies an intermediate position (SPEI, 0.432–0.838).

Similarly, VMDI exhibits the highest MI and lowest CE for SPI and SPTI. The MI values drop at the glacier-fed stations Gilgit (0.30) and Bunji (0.39), with CE values of 2.01 and 1.91, respectively, while JSDI shows MI = 0.48 and 0.63 and CE = 1.82 and 1.67, respectively, indicating better information retention for temperature-driven drought signals. VMDI’s lower-tail coefficients exceed 0.70 at all stations, reaching 0.89 at Gupis and 0.83 at Astore, indicating a strong capture of joint drought extremes. For SPEI, JSDI shows clear superiority at Gilgit and Bunji (lower tail, 0.560 and 0.590) compared with VMDI (0.226 and 0.262), reflecting JSDI’s advantage for evaporative extremes, where temperature and moisture availability are decoupled. VMDI achieves the lowest MAE in 17 of 18 cases: for SPI/SPTI, the MAE spans 0.139 to 0.375, a reduction of over 50% relative to JSDI (0.641–1.002) and MSDI (0.628–0.995). The Index of Agreement exceeds 0.93 in all SPI/SPTI cases, and the NSE reaches 0.970 (Astore–SPTI) and 0.948 (Chilas–SPTI). Most SPI/SPTI pairs have high KGE, except at Bunji and Gilgit, indicating a mismatch in variability that NSE does not penalize. The sole exception is Gilgit–SPEI, where JSDI’s MAE (1.005) slightly outperforms VMDI’s (1.014), but the difference is not statistically significant.

Extreme event detection confirms these patterns. VMDI’s F1 scores for SPI and SPTI range from 0.505 (Skardu–SPI) to 0.907 (Gilgit–SPTI), with $\textrm{DR} > 0.80$ at most stations. JSDI leads for SPEI at four stations, achieving F1 = 0.641 at Gupis with FAR = 0.000. At Skardu-SPTI, VMDI is the preferred index despite its lower F1 score, as JSDI’s FAR of 1.000 indicates no practical detection skill. The threshold-free AUC analysis corroborates these findings: VMDI–SPI/SPTI AUC ranges from 0.932 to 0.993, while the JSDI–SPEI AUC peaks at 0.907 (Gupis). The rankings remain consistent across the 10th–25th percentile drought definitions, confirming robustness. Temporal consistency is critical for operational use. The cross-correlation functions between VMDI and its constituents peak at lag 0 for all stations (VMDI–SPTI, r=0.971–0.986; VMDI–SPI, r=0.906–0.941), indicating no artificial smoothing. The lag sensitivity analysis indicates that a 1-month lag reduces the total vine dependence by 49.7% (Astore) to 79.2% (Bunji), validating the synchronous (zero-lag) combination. The smaller penalties at the glacier-dominated Astore and Skardu reflect snowmelt memory, while the larger penalties at Bunji and Gilgit indicate an instantaneous hydrological response.

The decay patterns also reveal distinct driver memories: SPTI and SPI exhibit short-term memory (sharp drops after lag 0), typical of the rapid response of semi-arid catchments to precipitation, whereas SPEI shows longer memory and seasonal oscillations, reflecting slower, temperature-driven processes such as snowmelt and evapotranspiration. This separation is essential for distinguishing precipitation-deficit from heat-driven droughts and for understanding their propagation through the hydrological system. Collectively, these results establish VMDI as a reliable index for precipitation-driven drought monitoring in the UIB, with JSDI serving as a valuable complement for temperature-driven evaporative extremes at stations such as Gilgit and Bunji. VMDI’s operational advantage lies in its fixed station-specific weights (SPEI, 0.227–0.283) and reliance on only three standard indices, enabling integration into early-warning systems without recalibration.

### Practical implementation of VMDI

VMDI relies on just three widely used indices and station-specific fixed weights, which facilitate its integration into existing monitoring systems. AUC analysis demonstrates that VMDI remains robust across the 10**–**25th percentile thresholds, accommodating different drought monitoring definitions. Future work should broaden VMDI to 3-, 6-, and 12-month accumulation scales, incorporate streamflow and soil moisture indices to represent hydrological drought propagation, and interpolate station weights spatially using covariates such as elevation, aridity, and glacial cover. Bias-adjusted projections under SSP2-4.5 and SSP5-8.5 could then be used to further evaluate future drought conditions over the UIB.

## Conclusion

This study presents substantive contributions to multivariate drought methodology by introducing a C-vine-copula-based index, VMDI, that consistently outperforms the existing composite indices (MSDI and JSDI), especially for precipitation-driven signals. Overall, VMDI–SPI/SPTI improves agreement by 15*–*30%, with MI gains > 41%, CE reduction of <29%, lower tail dependence >30%, MAE reductions > 50%, $\textrm{DR}> 33-37\%$, and AUC 0.932*–*0.993 across all stations compared with MSDI and JSDI. It ranks first across every evaluation metric for precipitation-driven drought signals. For evaporative extremes, JSDI serves as a valuable complement to VMDI; in snow- and glacier-fed catchments where temperature anomalies are decoupled from precipitation (Gilgit, Bunji), JSDI attains a substantial improvement over VMDI. Notably, these stations receive the lowest SPEI weights in VMDI (0.227–0.229), confirming that the vine-based weighting does not mask the decoupled signal but rather down-weights it appropriately. The vine-derived weights are physically consistent and temporally stable. The SPEI weights increase monotonically with glacier influence, from 0.227 at the lowest to 0.283 at the highest. Cross-correlations peak at lag 0 for all stations, and the lag sensitivity analysis confirms the optimality of the synchronous combination, with 1-month penalties ranging from 49.7% to 79.2%. The smaller penalties at the high-glacier stations reflect snow-melt memory, further validating the physical interpretability of the framework. VMDI is recommended for precipitation-dominated catchments, while JSDI is better suited for snow- and glacier-fed catchments where temperature-driven evaporative extremes dominate; both JSDI and VMDI provides the best results across all stations and the lag sensitivity for complex mountain basins.

## Supplementary Information

Below is the link to the electronic supplementary material.

Supplementary file 1 (pdf 399 KB)

## Data Availability

The data and code used in this paper are available to reproduce the results and can be obtained from the corresponding author upon reasonable request.
