# Supplementary material for: A novel multivariate vine-copula–based drought index with application to high-elevation catchments
Source: Environ Sci Pollut Res Int. 2026 Oct 1;33(32):16359–79. doi: 10.1007/s11356-026-38256-z (PMC13645877; doi:10.1007/s11356-026-38256-z)
Supplement: Supplementary file 1 [file 11356_2026_38256_MOESM1_ESM.pdf]

Supplementary Materials for: “A novel multivariate ine-copula based drought index with application to high-elevation catchments”

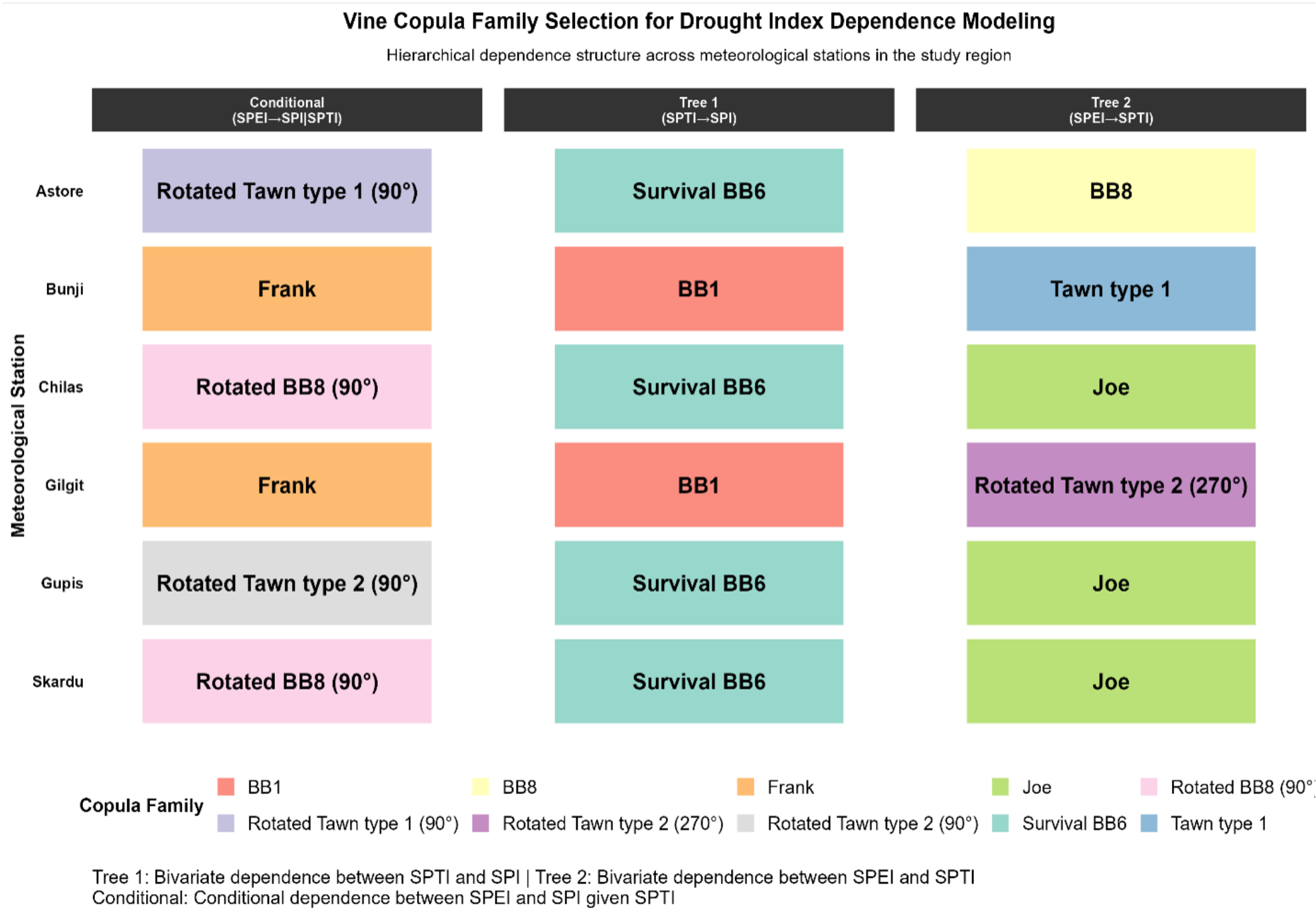

Fig. S.1: Schematic of the structure of the vine copula model to hierarchical dependence modelling of the drought indices (SPI, SPTI, SPEI) with station-specific configurations. The framework provides flexibility to represent tail dependence that would be of great importance in extreme drought analysis in heterogeneous hydroclimatic regimes.

Table S.1: Driver contribution percentages at each station. The table shows that in several cases, SPEI contributes more than 25% of the total signal, confirming that it is not redundant despite its lower average weight.

Station	SPTI (%)	SPI (%)	SPEI (%)
Gilgit	24.2	47.2	28.6
Skardu	18.6	39.2	42.2
Astore	31.2	33.8	35.0
Bunji	20.1	49.8	30.1
Chilas	16.5	45.0	38.5
Gupis	22.3	44.7	33.0

Table S.2: Spearman rank correlation (ρ) between the linear VMDI and the full vine-based Rosenblatt distance (a measure of multivariate extremeness), and the proportion of variance (R^2) captured by the linear approximation. All correlations are significant at $p < 0.001$.

Station	ρ	$R^2 = \rho^2$
Gilgit	0.866	0.749
Skardu	0.879	0.772
Astore	0.903	0.816
Bunji	0.861	0.741
Chilas	0.866	0.749
Gupis	0.856	0.733

Table S.3: Leave-One-Year-Out (LOYO) sensitivity analysis summary for all six stations. $f(c_{12})$ and $f(c_{13})$ represent the selection frequency of the optimal copula families for the SPTI-SPI and SPTI-SPEI pairs, respectively. ρ is the Spearman correlation, and RMSE is the root-mean-square error between the original VMDI and the LOYO-averaged VMDI.

Station	$SD(\omega_{SPTI})$	$SD(\omega_{SPI})$	$SD(\omega_{SPEI})$	$f(c_{12})$	$f(c_{13})$	ρ	RMSE
Gilgit	0.00018	0.00026	0.00020	1.000	1.000	0.900	0.012
Skardu	0.00029	0.00023	0.00019	0.998	1.000	1.000	0.015
Astore	0.00027	0.00022	0.00017	1.000	0.999	1.000	0.010
Bunji	0.00047	0.00063	0.00030	1.000	0.991	0.9999999	0.018
Chilas	0.00019	0.00026	0.00020	0.997	1.000	1.000	0.013
Gupis	0.00024	0.00030	0.00024	1.000	0.996	1.000	0.014

Table S.4: Statistical diagnostic test p-values for drought indices across stations

Station	Index	Ljung–Box	Runs	ADF	Shapiro–Wilk
Gilgit	SPI	2.23×10^{-10}	2.50×10^{-5}	0.01	2.38×10^{-11}
Skardu	SPI	4.15×10^{-8}	4.64×10^{-3}	0.01	4.46×10^{-11}
Astore	SPI	1.09×10^{-12}	1.39×10^{-10}	0.01	1.99×10^{-6}
Bunji	SPI	2.80×10^{-7}	2.46×10^{-2}	0.01	1.56×10^{-12}
Chilas	SPI	1.10×10^{-7}	4.74×10^{-6}	0.01	9.37×10^{-14}
Gupis	SPI	$< 1 \times 10^{-16}$	5.76×10^{-11}	0.01	1.37×10^{-16}
Gilgit	SPTI	1.84×10^{-6}	4.16×10^{-3}	0.01	5.77×10^{-11}
Skardu	SPTI	3.33×10^{-16}	6.59×10^{-7}	0.01	9.90×10^{-23}
Astore	SPTI	$< 1 \times 10^{-16}$	7.14×10^{-17}	0.01	1.83×10^{-8}
Bunji	SPTI	3.11×10^{-4}	2.38×10^{-1}	0.01	2.56×10^{-12}
Chilas	SPTI	7.73×10^{-8}	2.90×10^{-4}	0.01	2.81×10^{-14}
Gupis	SPTI	7.66×10^{-15}	2.36×10^{-6}	0.01	1.22×10^{-16}
Gilgit	SPEI	$< 1 \times 10^{-16}$	5.40×10^{-52}	0.01	3.34×10^{-5}
Skardu	SPEI	$< 1 \times 10^{-16}$	5.40×10^{-52}	0.01	2.83×10^{-5}
Astore	SPEI	$< 1 \times 10^{-16}$	1.74×10^{-44}	0.01	3.37×10^{-5}
Bunji	SPEI	$< 1 \times 10^{-16}$	1.60×10^{-45}	0.01	3.13×10^{-5}
Chilas	SPEI	$< 1 \times 10^{-16}$	5.40×10^{-52}	0.01	3.29×10^{-5}
Gupis	SPEI	$< 1 \times 10^{-16}$	1.06×10^{-48}	0.01	3.05×10^{-5}
